# Serological Insights into Infectious Agents Circulating in Lithuanian Goats

**DOI:** 10.3390/vetsci13010086

**Published:** 2026-01-15

**Authors:** Patricija Klibavičė, Tomas Kupčinskas, Saulius Petkevičius, Jūratė Buitkuvienė, Algirdas Šalomskas

**Affiliations:** 1 Department of Veterinary Pathobiology, Lithuanian University of Health Sciences, LT-44307 Kaunas, Lithuania; tomas.kupcinskas@lsmu.lt (T.K.); saulius.petkevicius@lsmu.lt (S.P.); algirdas.salomskas@lsmu.lt (A.Š.); 2 Serology Department, National Food and Veterinary Risk Assessment Institute, LT-08411 Vilnius, Lithuania; jurate.buitkuviene@nmvrvi.lt

**Keywords:** goats, caprine arthritis–encephalitis virus, *Toxoplasma gondii*, *Hypoderma* spp., *Neospora caninum*, *Mycoplasma agalactiae*, pestiviruses, Q fever, seroprevalence, mixed infections

## Abstract

Pathogens such as *Toxoplasma gondii*, lentiviruses (e.g., CAE), *Hypoderma* spp., *Neospora caninum*, *Mycoplasma* spp., and pestiviruses are important for goat farming in Lithuania; however, data on their prevalence remain limited. To address this gap, a multi-pathogen study was conducted between 2021 and 2024 using selected ELISA kits (ID.vet, Innovative Diagnostics, France). A total of 380 blood samples were collected from 30 goat herds across different regions of Lithuania; the sample size varied depending on the pathogen. Serum samples were tested for antibodies, and seroprevalence was calculated for each pathogen. The highest seroprevalence was detected for *T. gondii* (38.9%, 143/368) and CAE virus (19.5%, 74/380). Antibodies to *Mycoplasma* spp. (0.3%, 1/368), *Hypoderma* spp. (3.8%, 7/184), and *N. caninum* (0.5%, 2/368) were detected only sporadically, while no antibodies to Border disease virus or Q fever were identified. Mixed infections were found in 7.6% of samples. Chi-square analysis showed that co-infections with toxoplasmosis and CAE occurred more frequently than expected (χ^2^ = 19.05, *p* < 0.001). Herd size was significantly associated only with CAE seroprevalence (χ^2^ = 7.913, df = 1, *p* < 0.05). Overall, toxoplasmosis and CAE were identified as the most epidemiologically relevant infections in the Lithuanian goat population.

## Simple Summary

There is limited information in Lithuania regarding goat diseases and their epidemiological status. The aim of this study was to investigate the seroprevalence of major pathogens affecting goats, including *Toxoplasma gondii*, caprine arthritis–encephalitis virus (CAE), *Hypoderma* spp., *Neospora caninum*, *Mycoplasma* spp., pestiviruses (Border disease virus), and *Coxiella burnetii* (Q fever). A total of 380 blood samples were collected from goats in 30 herds across Lithuania. Antibodies against *Coxiella burnetii* and pestiviruses were not detected, whereas seropositivity to all other investigated pathogens was observed, indicating exposure within the goat population. Mixed infections were detected in 7.6% of the tested samples, with toxoplasmosis and CAE being the most frequent co-infections. The results provide insight into the epidemiological situation of important infectious diseases in the Lithuanian goat population. Continued surveillance, increased awareness among goat farmers, and further research are essential to improve herd health, prevent disease spread, and support sustainable goat farming in Lithuania.

## 1. Introduction

Goat farming in Lithuania plays an important role in the livestock sector, especially among small farms, where it is often considered an alternative to dairy or beef cattle farming. However, goat farming faces significant health challenges, particularly from infectious diseases. The spread of pathogens threatens animal health and productivity, may cause economic losses, and can affect animal welfare and human health. Despite the popularity of goat milk, limited veterinary surveillance and diagnostic resources have restricted studies on goat health [1,2,3].

Among the most common and important infectious diseases in the Lithuanian goat population, pathogens such as *Toxoplasma gondii*, lentiviruses (e.g., caprine arthritis–encephalitis (CAE) virus), *Hypoderma* spp., *Neospora caninum*, *Mycoplasma* spp., *Coxiella burnetii*, and pestiviruses (Border disease, BD) play key roles in goat health. *Toxoplasma gondii* causes reproductive disorders, mainly abortions, and is transmitted through ingestion of oocysts contaminating feed, water, or the environment. Small ruminant lentiviruses, such as the caprine arthritis–encephalitis virus, cause chronic inflammatory diseases including arthritis, mastitis, and encephalitis and are transmitted primarily via colostrum, milk, and close contact. *Hypoderma* spp. cause cutaneous myiasis and are transmitted by adult flies depositing eggs on the host. *Neospora caninum* leads to abortions and is transmitted both vertically and horizontally. *Mycoplasma* spp. cause mastitis, respiratory disease, and arthritis and spread through direct contact and milk. *Coxiella burnetii* causes Q fever, leading to reproductive disorders and posing a zoonotic risk; transmission occurs mainly via aerosols, birth products, and contaminated milk. Pestiviruses, such as Border disease virus, cause reproductive failure and congenital abnormalities and are transmitted through direct contact with infected animals. These infections negatively impact overall goat health and biosecurity, leading to reduced productivity and financial losses. Clinical manifestations vary, from asymptomatic infection to reproductive, respiratory, or nervous system disorders, as well as joint damage (e.g., arthritis, lameness) [2,4,5,6].

Mixed infections, where a goat is infected with more than one pathogen simultaneously, can complicate diagnosis, produce atypical clinical signs, and confound test results. They are also associated with more severe clinical signs and potentially greater economic losses. Control is challenging because pathogens differ in life cycles and drug resistance profiles. Management requires combined treatment approaches, vaccination, individualized deworming schedules, strict biosecurity, and continuous monitoring [7,8,9,10].

Despite the growth potential of goat farming, where both the number of registered goats and the average herd size have steadily increased over the past decade [11], Lithuania still lacks comprehensive data on prevalence, epidemiology, and disease impact. Previous localized studies on seasonal nematode infections and parasite resistance underscore the need for broader, country-level surveillance, especially for viral, protozoal, and bacterial infections. Analyzing infectious diseases in the goat population is therefore essential for developing effective prevention and control strategies.

The aim of this article is to examine the seroprevalence and co-infections of major infectious diseases, including toxoplasmosis, lentiviral infections, hypodermosis, neosporosis, mycoplasmosis, Q fever, and pestivirus infections, in the Lithuanian goat population, to evaluate the current situation and identify potential associations between these infections.

## 2. Materials and Methods

Permission to conduct this research was obtained from the Bioethics Committee (Approval No. 2024-BEC3-T-008, 12 March 2024). All procedures were carried out in accordance with relevant ethical guidelines and regulations governing animal research.

### 2.1. Data Source

**Figure 1:**
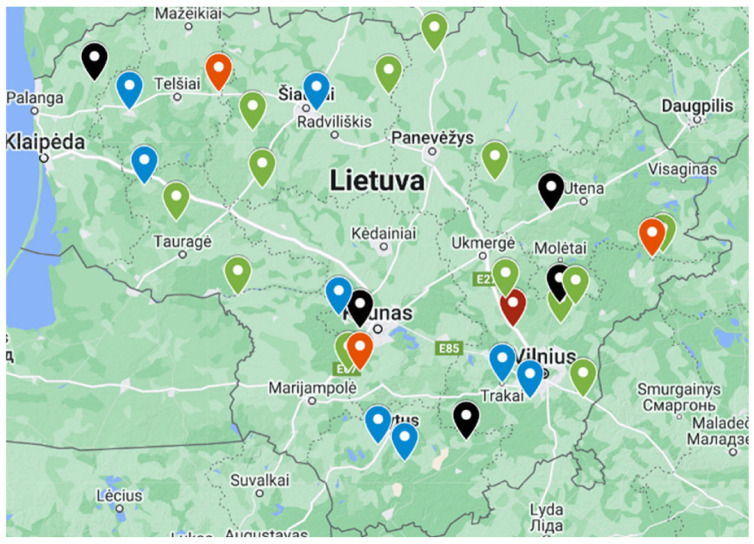
Map of visited goat farms during study in Lithuania, 2021–2024 (colors in the map are not related to the results of the study).

To assess the infection status of goats in Lithuania, a comprehensive study covering multiple pathogens was conducted from 2021 to 2024. Goat farmers were invited to participate voluntarily through online invitations and registration forms. From the submitted forms, farmers keeping at least 10 goats were selected to ensure sufficient sampling per herd. Information provided by participating farmers indicated that feeding practices and management conditions were broadly comparable across the included herds, reflecting typical semi-intensive goat production systems in Lithuania. In total, 30 goat farms from various regions of Lithuania were visited (Figure 1).

Herds of various sizes participated in this research, ranging from 10 to 500 goats. Considering the studied herd sizes and the average goat herd size in Lithuania, the herds were categorized as small (10–20 goats, *n* = 15), medium (21–30 goats, *n* = 6), and large (>30 goats, *n* = 9) to estimate goat morbidity rates according to herd size. This categorization was based on the size of the studied herds in Lithuania and on previous studies [12].

### 2.2. Blood Sample Collection and Testing

Goats for testing were selected randomly or based on the presence of clinical symptoms of infection. From each farm, 10–15 whole blood samples were collected from the jugular vein using blood collection tubes with clot activator. For serum separation, the samples were centrifuged at 1500 rpm for 15 min. Separated serum was aliquoted into cryotubes and stored at −20 °C until testing. In total, 380 blood serum samples were tested for *CAEV* and toxoplasmosis, 368 samples for Q fever, border disease, mycoplasmosis, and neosporosis, and 184 samples for hypodermosis.

Serum samples were tested for pathogen-specific antibodies using commercially available enzyme-linked immunosorbent assay (ELISA) kits (Innovative Diagnostics, Grabels, France) Antibodies against *Toxoplasma gondii* were detected using the ID Screen^®^ Toxoplasmosis Indirect Multi-species ELISA; antibodies against small ruminant lentiviruses (caprine arthritis–encephalitis virus/maedi–visna virus, CAEV/MVV) using the ID Screen^®^ MVV/CAEV Indirect Screening Test; antibodies against pestiviruses (bovine viral diarrhea virus/Border disease virus) using the ID Screen^®^ BVD p80 Antibody Competition ELISA; antibodies against *Neospora caninum* using the ID Screen^®^ Neospora caninum Indirect ELISA; antibodies against *Hypoderma* spp. using the ID Screen^®^ Hypodermosis Indirect ELISA; antibodies against *Coxiella burnetii* using the ID Screen^®^ Q Fever Indirect Multi-species ELISA; and antibodies against *Mycoplasma agalactiae* using the ID Screen^®^ Mycoplasma agalactiae Indirect ELISA.

For each ELISA kit, the specific antigen(s) used for coating the plates were as follows: *Toxoplasma gondii* P30 antigen for *T. gondii* serology; a panel of peptides derived from MVV/CAEV TM, gp135 and p25 proteins for lentivirus serology; the non-structural p80 (NS3) protein for pestivirus (BVD/BD) antibody detection; a purified *Neospora caninum* extract for *N. caninum* serology; a *Coxiella burnetii* antigen from a bovine isolate for Q fever serology; and a recombinant *Mycoplasma agalactiae* P48 protein for *M. agalactiae* antibody detection. The antigen used for *Hypoderma* spp. serology was the corresponding species-specific extract provided in the kit.

All assays were conducted strictly according to the manufacturer’s instructions, including recommended serum dilutions, incubation times, washing steps, and substrate reactions. Each ELISA kit included manufacturer-provided positive and negative control sera, which were run on every plate to validate assay performance. Only assay runs in which the control values met the acceptance criteria specified by the manufacturer were considered valid.

The catalog numbers for the ELISA kits used were as follows: ID Screen^®^ *Toxoplasma gondii* Indirect Multi-species (Cat. No. TOXOS MS 2P), ID Screen^®^ MVV/CAEV Indirect Screening Test (Cat. No. VISNAS 5P), ID Screen^®^ BVD p80 Antibody Competition (Cat. No. BVDC-5P), ID Screen^®^ *Neospora caninum* Indirect (Cat. No. NCS 5P), ID Screen^®^ *Hypoderma* spp. Indirect (Cat. No. HYPOS-2P), ID Screen^®^ Q fever Indirect Multi-species (Cat. No. FQS MS 5P), and ID Screen^®^ *Mycoplasma agalactiae* Indirect (Cat. No. MAGALS 5P).

Optical density (OD) values were measured at 450 nm with background correction at 620 nm using a BioTek Synergy MX Microplate Reader spectrophotometer (BioTek Instruments, Winooski, VT, USA). The presence or absence of antibodies in each serum sample was determined by comparing the sample OD with the kit-provided controls. Cut-off values were calculated according to the formulas and interpretation criteria specified by the manufacturer for each assay, allowing classification of samples as positive, negative, or doubtful. True positive and true negative results were confirmed based on compliance with validated control ranges and cut-off thresholds provided by the assay manufacturer, minimizing the risk of false-positive or false-negative results.

Representative ELISA results for all pathogens are shown in Supplementary Tables S1–S7.

### 2.3. Data Management and Statistical Analysis

All data from the survey and the results of serological tests were recorded in a database and used for analysis. Statistical analyses were performed using IBM SPSS Statistics software, version 30.0.0 (IBM Corp., Armonk, NY, USA) and Pearson’s Chi-square (χ^2^) tests were calculated. The level of significance was set at *p* < 0.05 for all analyses.

The confidence interval (CI) of seroprevalence in the goat population and the reliability of differences in prevalence percentages at a 95% probability were calculated using the EpiTools epidemiological calculator program (https://epitools.ausvet.com.au/, accessed on 31 December 2024) according to the formula:

CI = *p* ± Z × σ, (1)

where CI is the confidence interval, *p* is the proportion of positive samples, Z is the confidence coefficient (1.96 for 95% confidence level), and σ is the standard error.

### 2.4. Clarification: Inclusion of All Diseases and p-Values

All seven tested diseases were included in the chi-square analysis. Although some diseases had smaller sample sizes or no positive results (e.g., Q fever and Border disease), the χ^2^ statistics were calculated relative to the number of animals tested for each pathogen, ensuring comparability across pathogens with unequal sample sizes. Diseases with zero positives but high expected values (such as Q fever) still contributed to the overall statistical outcome.

## 3. Results

### 3.1. Study Population

**Figure 2:**
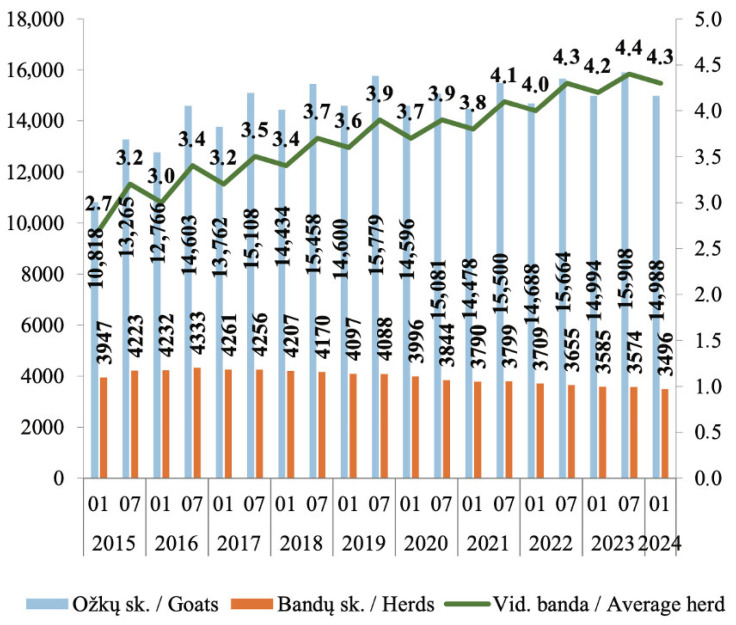
Goats herd number dynamics 2015–2024. Blue color shows a total number of goats, orange color—number of herds and green color—number of average herd size. Source: [11].

According to statistics from national institutions, the number of registered goats increased from 10,818 to 14,988 between the beginning of 2015 and the beginning of 2024. The number of goat herds remained largely stable during this period. Currently, in 2025, 3191 goat herds and 14,160 goats are registered by the Lithuanian Agricultural Data Center. The average herd size has also increased, rising from 2.7 to 4.3 animals per herd over the same period [11] (Figure 2).

### 3.2. Prevalence

#### 3.2.1. Prevalence of *Toxoplasma* spp.

**Figure 3:**
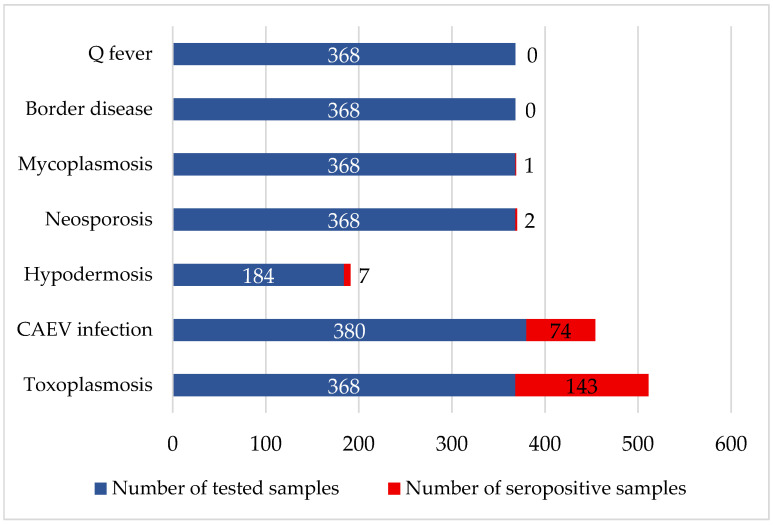
Seroprevalence of different pathogens in tested goats. *X*-axis: number of samples; *Y*-axis: tested disease.

This study assessed the prevalence of toxoplasmosis caused by *Toxoplasma gondii* in the Lithuanian goat population. A total of 368 goat blood serum samples were tested. Antibodies against *T. gondii* were detected in 38.9% of samples. A 95% confidence interval (CI) was calculated, with values ranging from 33.85% to 44.05% (Figure 3). At the farm level, *Toxoplasma gondii* antibodies were found in 29 of 30 visited goat farms (96.6%; 95% CI 82.78–99.92%).

#### 3.2.2. Prevalence of Lentivirus (CAEV)

Caprine arthritis–encephalitis virus (CAEV) infection was assessed as part of the broader panel of pathogens studied. Herds were considered infected if at least one goat in the herd tested positive. A total of 380 goat blood serum samples were collected. Antibodies against CAEV were detected in 19.5% of samples. The 95% confidence interval (CI) of this prevalence ranged from 15.61% to 23.82% (Figure 3). Seroprevalence of CAEV varied significantly between farms. In 17 of 30 investigated goat farms (56.7%; 95% CI 37.43–74.54%), at least one seropositive animal was found.

#### 3.2.3. Prevalence of *Hypoderma* spp.

During the study, serological tests for *Hypoderma* spp. infection were performed to determine the prevalence of antibodies among goats. A total of 184 blood serum samples were tested. Antibodies against *Hypoderma* spp. were detected in 3.8% of all tested samples. The 95% confidence interval (CI) for this prevalence ranged from 1.54% to 7.68% (Figure 3). Additionally, *Hypoderma* spp. were tested in 16 goat farms, and at least one seropositive sample was found in only 5 farms (29.4%; 95% CI 11.02–58.66%).

#### 3.2.4. Prevalence of *Neospora caninum*

An analysis of the prevalence of *Neospora caninum* antibodies among goats kept in Lithuania was performed. A total of 368 goat blood serum samples were tested, of which 0.5% were positive for *N. caninum* antibodies. The 95% confidence interval (CI) for this prevalence was 0.07–1.95% (Figure 3). At the farm level, 30 goat farms were tested, and only 2 farms (6.7%; 95% CI 0.81–22.07%) had at least one positive case.

#### 3.2.5. Prevalence of *Mycoplasma agalactiae*

In this study, the prevalence of *Mycoplasma agalactiae* antibodies in the Lithuanian goat population was assessed. A total of 368 blood serum samples were collected. Only 0.3% of samples tested positive (95% CI 0.01–1.51%; Figure 3). At the farm level, one of 30 farms (3.3%; 95% CI 0.08–17.22%) had at least one positive goat.

#### 3.2.6. Prevalence of Pestivirus and *Coxiella burnetii*

Serological testing of 368 goat serum samples revealed no antibodies against pestiviruses or *Coxiella burnetii* (Figure 3), suggesting that infection with these pathogens was absent or at a very low level in the study population.

### 3.3. Chi-Square Contribution by Disease

The breakdown of the chi-square contributions reveals that Toxoplasma gondii is the main driver of statistical deviation, contributing over 65% of the total χ^2^ value. This is because its observed positives (143) were significantly higher than expected (approximately 35).

CAE (lentivirus) also showed a higher-than-expected number of positives, reinforcing its importance as a secondary contributor. Conversely, diseases such as *Coxiella burnetii* (Q fever), Border disease, mycoplasmosis, and *Neospora caninum* (neosporosis) had no or very few positives, despite higher expected counts. These large gaps between observed and expected values also contributed significantly to the chi-square statistic.

In summary, almost all diseases showed statistically significant differences between observed and expected values, confirming that the distribution of seropositive cases is far from uniform and reflects actual differences in prevalence across the goat population (Table 1).

### 3.4. Pairwise Statistical Comparison and Association Analysis of Infectious Agents

This section presents all pairwise comparisons between the tested diseases using three statistical methods: the Z-test for comparing proportions (seroprevalence), Fisher’s exact test for binary associations (particularly suitable for rare events), and the Chi-square test for co-occurrence associations. Each row compares two diseases and indicates whether the difference or association is statistically significant (Table 2).

### 3.5. Mixed Infections

During the study of various goat diseases, some serum samples collected from all tested goats were positive for more than one pathogen. Antibodies against a single disease were found in 171 samples, almost all of which were positive for toxoplasmosis. Antibodies against two diseases were detected in 28 samples, with co-infections of toxoplasmosis and CAE virus being the most common and the only statistically significant combination. Antibodies against three diseases were found in one sample, corresponding to toxoplasmosis, neosporosis, and CAE virus infection. These findings emphasize the importance of monitoring co-infections in herd health management, particularly for diseases with a higher overlap, such as toxoplasmosis and CAE (Table 3).

### 3.6. Association Test Based on Mixed Infections

This section presents the chi-square association test results between pairs of diseases based only on goats with confirmed infections (*n* = 200). It evaluates whether infections tend to co-occur in the same individuals more often than expected by chance. The analysis focused on whether two diseases are statistically associated within co-infected animals.

The results show that only the pair toxoplasmosis + CAE had a statistically significant association (χ^2^ = 19.05, *p* < 0.001), indicating a higher-than-expected co-occurrence.

All other disease pairs either showed no significant association or had insufficient data for meaningful testing, particularly for rare infections such as mycoplasmosis, neosporosis, and hypodermosis.

### 3.7. The Distribution of Disease in Different Size of the Herd

#### 3.7.1. Caprine Arthritis–Encephalitis

**Figure 4:**
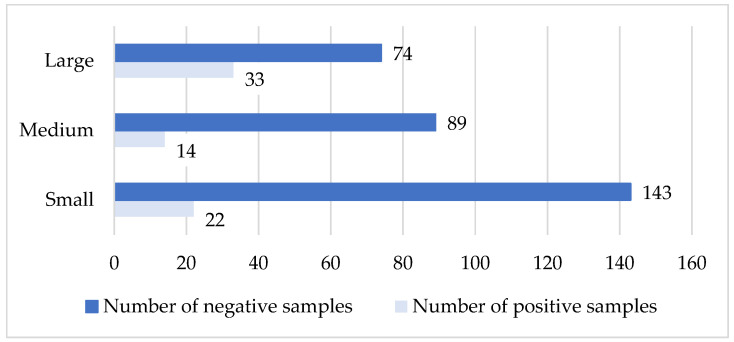
Comparison of sample status with the size of the herd (CAE virus). *X*-axis: number of tested samples; *Y*-axis: type of farm according to size.

This analysis evaluates the distribution of seropositive and seronegative samples for caprine arthritis–encephalitis across 30 goat herds, including small (15/30), medium (6/30), and large (9/30) herds. A total of 380 blood samples were tested. The results showed a similar distribution in small and medium herds (small: 13% positive, 87% negative; medium: 14% positive, 86% negative), while in large herds the distribution differed more markedly (33% positive, 67% negative). Analysis of seropositive sample frequencies revealed a statistically significant difference between herd sizes (χ^2^ = 7.913, df = 1, *p* < 0.05, Figure 4). It should be noted that the small sample sizes in some herd size categories may limit statistical power; therefore, subtle differences could remain undetected.

#### 3.7.2. Hypodermosis

**Figure 5:**
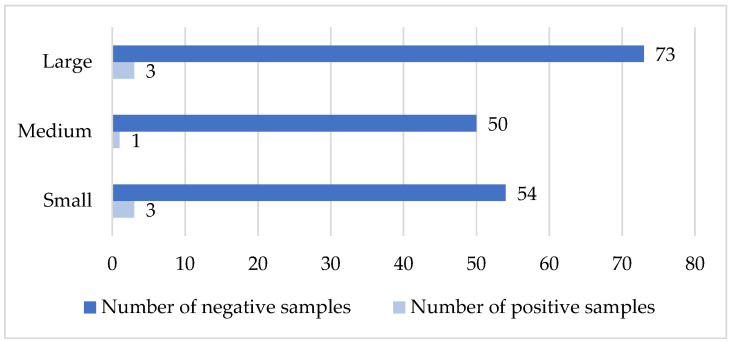
Comparison of sample status with the size of the herd (hypodermosis). *X*-axis: number of tested samples; *Y*-axis: type of farm according to size.

The total number of tested samples was 184. The prevalence of seropositive and seronegative samples in herds of different sizes was as follows: small herds, 5.3% positive and 94.7% negative; medium herds, 2% positive and 98% negative; large herds, 4% positive and 96% negative. Analysis of seropositive sample frequencies revealed no statistically significant association between herd size and seroprevalence for hypodermosis (χ^2^ = 0.805, df = 2, *p* > 0.05, Figure 5).

#### 3.7.3. Toxoplasmosis

**Figure 6:**
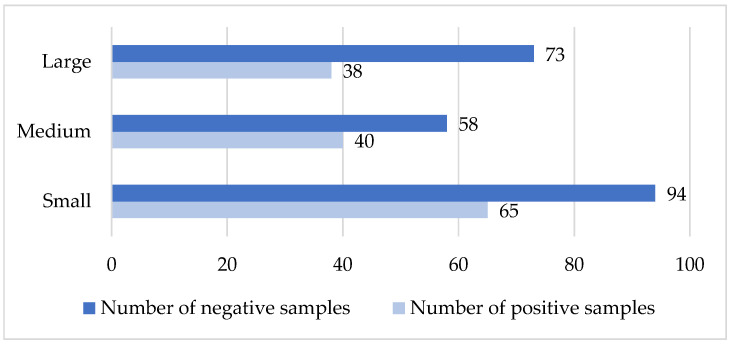
Comparison of sample status with the size of the herd (toxoplasmosis). *X*-axis: number of tested samples; *Y*-axis: type of farm according to size.

The total number of samples tested was 368. The distribution of seropositive and seronegative samples across herds of different sizes showed almost the same pattern in small and medium herds (small herds: 40.9% positive and 59.1% negative; medium herds: 40.8% positive and 59.2% negative), while in large herds the distribution showed lower frequency differences (4.2% positive and 65.8% negative). Analysis of seropositive sample frequencies revealed no statistically significant association between herd size and seropositivity for toxoplasmosis (χ^2^ = 1.431, df = 2, *p* > 0.05, Figure 6).

#### 3.7.4. Neosporosis and Mycoplasmosis

**Figure 7:**
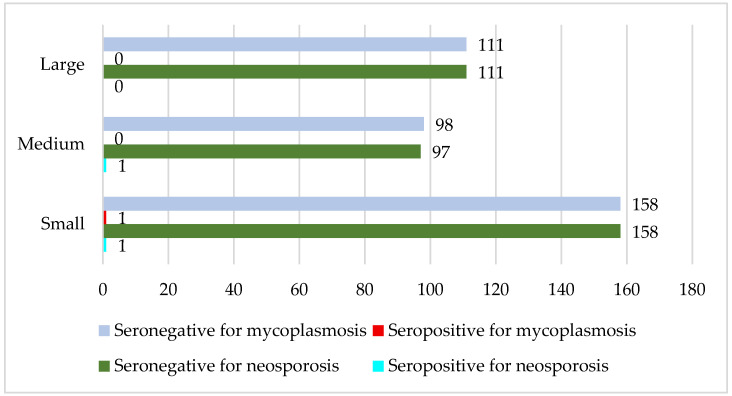
Comparison of sample status with the size of the herd (neosporosis and mycoplasmosis). *X*-axis: number of tested samples; *Y*-axis: type of farm according to size.

The total number of samples tested was 368. The prevalence of seropositive and seronegative samples across herds of different sizes was as follows: small herds—0.6% positive and 99.4% negative for neosporosis and mycoplasmosis; medium herds—1% positive and 99% negative for neosporosis, and 0% positive and 100% negative for mycoplasmosis; large herds—0% positive and 100% negative for both diseases. Analysis of seropositive sample frequencies revealed no statistically significant association between herd size and seropositivity for neosporosis (χ^2^ = 1.040, df = 2, *p* > 0.05) and mycoplasmosis (χ^2^ = 1, df = 2, *p* > 0.05, Figure 7).

## 4. Discussion

Environmental factors can influence the spread of infectious diseases in goats. For example, Toxoplasma gondii oocysts survive better under warmer and wetter conditions, increasing the risk of contamination of soil, water, and feed. Urbanization and growing cat populations, including stray cats, may further contribute to parasite transmission [13,14]. In contrast, climate factors do not directly affect CAE virus; however, heat stress can compromise the immune system, making goats more susceptible to infections and increasing the likelihood of latent infections becoming clinical (e.g., arthritis). Heat stress can also reduce milk production and alter milk composition [15,16,17]. Additionally, small herd sizes and limited resources may restrict farmers’ ability to maintain optimal biosecurity under challenging environmental conditions [18,19]. These observations may be relevant to our study and underscore the importance of vigilant disease surveillance, improved management programs, and close attention to goat health.

During our research, serological tests revealed that, among the infectious diseases of goats tested, toxoplasmosis and caprine arthritis–encephalitis (CAE) were the most common. Meanwhile, mycoplasmosis, hypodermosis and neosporosis were detected in isolated cases. Border disease and Q fever were not detected in any of the samples.

Toxoplasmosis showed the highest seroprevalence among all the diseases tested, with 38.9% of the goats positive for *Toxoplasma gondii*, indicating wide circulation of this parasite in the herds. Similar levels of seropositivity have been reported in Switzerland (50.5%) and Spain (48%) [20,21]. For example, a recent study in goat populations highlighted that *Toxoplasma gondii* remains one of the most prevalent pathogens worldwide, with seroprevalence estimates comparable to those observed in the present study [22]. This supports the notion of widespread circulation of *T. gondii* among small ruminants and underscores the global relevance of our findings. A meta-analysis (2022) indicated that 31.8% of goats are seropositive globally, with European countries reporting ranges between 28% and 70%. For instance, in Poland in 2018, seroprevalence exceeded 36.8%, while in Greece it was approximately 28% [23,24,25,26]. Our results of nearly 39% seropositivity highlight the high spread of infection at both the individual animal and farm levels, emphasizing the risk of abortions and zoonotic transmission. As toxoplasmosis in goats is often subclinical, this study focused on serological evidence of exposure rather than clinical disease. Clinical outcomes and farm-level histories were not systematically recorded; therefore, no direct association between seroprevalence and clinical manifestations could be assessed. Toxoplasmosisis considered endemic in the Lithuanian goat population, with seropositive animals reported over 20 years ago, reaching rates above 50% in some cases and even exceeding 80% in others. These findings underscore the need to further analyze infection routes and implement effective control measures on farms [27].

Caprine arthritis–encephalitis (CAE), although a slowly progressive disease, is of great epidemiological importance. The 19.5% seropositivity rate found in the study indicates a wide spread of this disease among goat herds, which is in line with data from other countries; for example, in Albania, a prevalence of up to 33.5% has been reported in dairy herds [28]. Since CAE virus is often spread through infected milk or colostrum, which is the primary route, seropositivity may be related to insufficient isolation of young goats after birth from seropositive ones. Unfortunately, despite main control measures, CAE virus also spreads via horizontal transmission through close contact between herd goats [12,29].

Our study revealed the current seroprevalence of small ruminant lentivirus infection in Lithuanian goat herds. Caprine arthritis–encephalitis was diagnosed in more than half of the tested herds (17 of 30), and the seroprevalence was 19.5% (95% CI, 15.6–23.8%) of all tested animals. Although the level of seropositivity in individual animals is low, the infection is relatively widely distributed on a farm scale, and CAE disease is endemic in Lithuania. This may indicate the prevalence of isolated cases or possible foci of infection within herds, and further studies are required to monitor and control CAE virus in the Lithuanian goat population. Furthermore, researchers in Poland have conducted extensive studies on CAE disease. Their latest study included 165 herds across various regions of Poland and tested 8354 adult goats. It was found that 60% of tested herds were seropositive. These results show that seropositivity at the herd level is similar to the results obtained in our study, with the prevalence at the herd level in Lithuania reaching almost 57% [12,29,30].

Hypodermosis, caused by *Hypoderma* larvae, was found in 3.8% of goats tested. Although this disease is not widespread in Lithuania and the level of seropositivity in individual animals is low, isolated cases indicate that the parasite is still present, and a possible increase in disease cases could occur in the future. This emphasizes the importance of vigilance in differential diagnosis.

A systematic review in 2020 reported an average seroprevalence of neosporosis among goats of approximately 5.99% (95% CI, 4.38–7.83%) [24]. Our results for neosporosis (0.5%) are significantly lower than the global average, but they confirm that the infection still exists in the Lithuanian goat population, albeit in isolated cases. Another regional study in Turkey reported a seroprevalence of 4.89% among 184 goats, and also showed a correlation with reproductive disorders, although the difference between aborted and non-aborted groups was not statistically significant [26]. In conclusion, our results indicate a low prevalence of *Neospora caninum* infection among the goats and farms included in the study. The low prevalence of *N. caninum* infection in Lithuania may be associated with limited contact between dogs and herds and more intensive biosecurity measures, whereas in Turkey the spread of this infection is likely influenced by traditional grazing practices alongside sheepdogs and a higher prevalence of infection sources in the environment. However, given the relatively wide confidence intervals, further studies with larger sample sizes would help to more accurately determine the prevalence of this infection in the wider goat population.

The incidence of mycoplasmosis (0.3%) may reflect the low prevalence of this bacterial infection, but this does not mean that it is of no clinical importance. *Mycoplasma agalactiae* causes mastitis, arthritis, respiratory distress, keratoconjunctivitis, and even reproductive issues. This infection in goats often presents with non-specific symptoms, especially in early or mild cases. Low seropositivity in a herd does not rule out infection—carriers may not show strong antibody responses or clinical signs [31]. The detection of a single positive sample highlights the need for continuous epidemiological monitoring (especially in mixed herds) and preventive measures to limit the spread of the disease.

The observed incidence of mycoplasmosis (0.3%) and hypodermosis (3.8%) indicates that these pathologies exist, even sporadically. This emphasizes the importance of vigilance in differential diagnosis.

Border disease and Q fever were not detected in any of the samples in this study. This may indicate effective biosecurity practices or a low prevalence of infections in the region. However, given the zoonotic risk of Q fever, continuous monitoring and strict registration of reproductive disorders remain important [32,33]. The results obtained during the study are in line with studies in other Baltic countries (e.g., Latvia, Estonia)—Q fever is practically undetectable in goats, and cases of Border disease have become extremely rare. Moreover, Q fever has recently been reported in neighboring Poland, highlighting the need for ongoing surveillance in the region [34].

The pairwise statistical analysis (Z-Test, Fisher Test, and Chi-square association) revealed several important patterns among the diseases tested in goats. *Toxoplasma gondii* showed consistently and significantly higher prevalence than all other diseases. It was involved in most of the statistically significant differences (Z-tests) and also showed significant co-occurrence with CAE (Caprine arthritis–encephalitis virus), suggesting possible shared risk factors or widespread exposure. CAE (Caprine arthritis–encephalitis virus) was the second most common infection and showed strong statistical differences in prevalence when compared to rarer diseases (e.g., *Neospora caninum*, *Mycoplasma agalactiae*, Border disease). It also demonstrated significant association with *T. gondii*, indicating a tendency for co-infection. *Hypoderma* spp. had a relatively low prevalence but still showed statistically significant differences in comparison to highly prevalent diseases. It was weakly associated with *T. gondii* in mixed infections. *Neospora caninum* and *Mycoplasma agalactiae* were extremely rare, with only 2 and 1 positive samples, respectively. This rarity makes statistical interpretation difficult. Although they had high odds ratios when compared to other diseases, the *p*-values in Fisher’s and Chi-square tests were not significant, indicating no reliable evidence of association. Q fever and Border disease had no detected positives, which led to undefined or non-significant test statistics in many pairwise comparisons. Their low detected seroprevalence may reflect either true rarity in the population or limitations in detection, particularly the small number of samples tested for these pathogens. Most statistically significant results (*p* < 0.05) were due to comparisons involving high-prevalence diseases, such as *T. gondii* and CAE, against rare or undetected diseases. While Fisher’s exact test was less sensitive due to the small sample sizes, it provided a valuable check for these low-frequency comparisons.

During serological tests for different infectious diseases, it was found that some goats were seropositive for multiple infections. A total of 171 samples (out of all tested) were positive for at least one disease. Of these, 116 samples were seropositive only for *Toxoplasma gondii* (out of 368 tested). Additionally, 28 samples had seroprevalence for two diseases, of which 19 were *T. gondii* and CAEV infection (368 and 380 goats tested), and only 1 sample was seropositive for three diseases—*T. gondii*, *Neospora caninum*, and CAEV infection.

Mixed seropositivity accounted for 7.6% of all samples tested (29 out of 380 tested for at least one disease), which is a significant proportion. *Toxoplasma gondii* and CAE were particularly common together, suggesting a possible link between environmental hygiene, immune status, and infection transmission mechanisms. Similar results have been obtained in other studies. For example, in Ecuadorian farms, 7.7% of goats were seropositive for three diseases simultaneously, most often a combination of *T. gondii*, *N. caninum*, and *Cryptosporidium* spp. [35]. Another study indicated that exposure to two or more pathogens may be associated with environmental and regional factors [36].

Although specific co-infection data for *T. gondii* and *N. caninum* in Lithuanian goats remain unavailable, studies from neighboring Poland show that goats often harbor both parasites concurrently (e.g., 100% herd-level seroprevalence for *T. gondii* and ~9% for *N. caninum*). Given similar environmental conditions, mixed protozoal infections may also occur in Lithuania [24].

In the study, toxoplasmosis accounted for 67.8% of all single-disease cases (116/171) and was included in almost all mixed infection combinations. This is consistent with the literature, as *T. gondii* remains one of the most common parasitic zoonoses in goats, with a high potential for interaction with other infections [37].

Seropositivity for three diseases—*T. gondii*, *N. caninum*, and CAE virus—in a single sample may reflect a complex infectious background in an individual animal. Such a multi-pathogenic profile may have a greater impact on reproduction, immunity, and general health. Studies show that such cases are not rare in intensive farming conditions [38].

Analysis of the distribution of diseases across different herd sizes showed that only caprine arthritis–encephalitis (CAE) had a statistically significant association with herd size (χ^2^ = 7.913, df = 1, *p* < 0.05). However, lower prevalence in smaller herds is not guaranteed. Although some studies show lower overall CAEV prevalence in smaller herds, others describe very high herd prevalence, suggesting that the infection may still be prevalent in small herds [39,40].

Analysis of seropositive samples revealed no statistically significant association between herd size and seropositivity for hypodermosis, toxoplasmosis, neosporosis, and mycoplasmosis (*p* > 0.05). Differences in prevalence among herd sizes are likely due to random variation rather than herd size effect. The distribution of seropositive versus seronegative cases does not differ sufficiently across small, medium, and large herds to conclude that herd size is related to infection risk. Positive case numbers are very low, reducing the statistical power to detect real differences. Regarding toxoplasmosis, seroprevalence tends to decrease with increasing herd size, but this is not always the case and may depend on factors such as farming system, animal age, and presence of cats. Some studies describe higher risk in larger or intensive farms due to increased animal density, while others report higher prevalence in smaller or extensive systems [41,42,43].

According to published literature, herd size may influence the seroprevalence of diseases in goats, although the relationship is complex and depends on other factors. Some studies have linked herd size to the seroprevalence of hypodermosis in goats, suggesting that larger herds may be at higher risk due to higher animal density or management practices [5]. A study in Brazil found that herds with 51–100 goats had a significantly higher likelihood of M. agalactiae infection compared to smaller herds (≤50 goats), with an odds ratio of 7.1 (95% CI: 2.4–20.6) [44]. This may be due to increased animal movement, higher introduction rates of new animals, and challenges in maintaining biosecurity in larger herds. For neosporosis, studies indicate that larger herds may increase infection risk, but smaller herd sizes may also matter, particularly if farms have more dogs [45,46].

The prevalence of Border disease and Q fever across herd sizes was not analyzed due to the absence of positive cases in tested samples. Studies suggest higher risk of Q fever in larger herds due to increased opportunities for pathogen introduction and transmission. The effect of herd size on Border disease seroprevalence is less clear, depending on multiple factors. One study found no significant association with herd size but noted a strong link with the presence of cattle on the same farm [47,48].

## 5. Conclusions

Toxoplasmosis and caprine arthritis–encephalitis (CAE) are the most epidemiologically relevant diseases in this population. The very low prevalence of Neospora caninum, Mycoplasma agalactiae, Q fever, and Border disease suggests that these pathogens are either not currently circulating at meaningful levels or were missed due to limitations in sampling or test sensitivity.

Mixed infections were observed in approximately 7.6% of samples, with toxoplasmosis being the most frequent component, highlighting their potential clinical relevance and impact on productivity. Analysis of herd size revealed a statistically significant association only for CAE, while no significant relationship was found between herd size and seropositivity for hypodermosis, toxoplasmosis, neosporosis, or mycoplasmosis (*p* > 0.05).

Overall, these results emphasize the importance of targeted monitoring and control measures, particularly for the most prevalent and epidemiologically significant diseases, to reduce the risk of spread and maintain herd health. Recommended strategies include combining diagnostic methods (e.g., ELISA testing for multiple pathogens simultaneously) to strengthen biosecurity, regulating feed contamination, limiting contact with other animals (cats, dogs), improving colostrum management, and incorporating regular monitoring of mixed infections into farm health programs.

## Figures and Tables

**Table 1:** Observed, expected values, chi-square contribution, and associated *p*-value for each disease.

Disease	Observed Positives	Expected Positives	Chi-Square Contribution	*p*-Value
Toxoplasmosis	143	34.75	337.23	0.000
CAE (lentivirus)	74	35.88	40.49	1.972 × 10^−10^
Hypodermosis	7	17.37	6.19	1.281 × 10^−2^
Neosporosis	2	34.75	30.86	2.768 × 10^−8^
Mycoplasmosis	1	34.75	32.78	1.033 × 10^−8^
Q fever	0	34.75	34.75	3.751 × 10^−9^
Border disease	0	34.75	34.75	3.751 × 10^−9^

**Table 2:** Pairwise comparison of diseases.

Disease 1	Disease 2	Significant (*p* < 0.05)	Tests with Significant Result	Significant Difference	Significant Association
Toxoplasmosis	Cae (lentivirus)	Yes	Z-test, fisher, chi^2^	Yes	Yes
Toxoplasmosis	Hypodermosis	Yes	Z-test, fisher, chi^2^	Yes	Yes
Toxoplasmosis	Neosporosis	Yes	Z-test	Yes	No
Toxoplasmosis	Mycoplasmosis	Yes	Z-test	Yes	No
Toxoplasmosis	Q fever	Yes	Z-test	Yes	No
Toxoplasmosis	Border disease	Yes	Z-test	Yes	No
Cae (lentivirus)	Hypodermosis	Yes	Z-test, fisher, chi^2^	Yes	Yes
Cae (lentivirus)	Neosporosis	Yes	Z-test, fisher, chi^2^	Yes	Yes
Cae (lentivirus)	Mycoplasmosis	Yes	Z-test	Yes	No
Cae (lentivirus)	Q fever	Yes	Z-test	Yes	No
Cae (lentivirus)	Border disease	Yes	Z-test	Yes	No
Hypodermosis	Neosporosis	Yes	Z-test, fisher, chi^2^	Yes	Yes
Hypodermosis	Mycoplasmosis	Yes	Z-test, fisher, chi^2^	Yes	Yes
Hypodermosis	Q fever	Yes	Z-test	Yes	No
Hypodermosis	Border disease	Yes	Z-test	Yes	No
Neosporosis	Mycoplasmosis	Yes	Fisher, chi^2^	No	Yes
Neosporosis	Q fever	No	None	No	No
Neosporosis	Border disease	No	None	No	No
Mycoplasmosis	Q fever	No	None	No	No
Mycoplasmosis	Border disease	No	None	No	No
Q fever	Border disease	No	None	No	No

Note: the frequencies are calculated from the total number of samples (380), as the analysis is based on the context of the entire sample. Some infections (hypodermosis—184 samples) were tested in a smaller sample, therefore the frequencies are estimated with caution.

**Table 3:** The occurrence of antibodies against the studied disease and their co-occurrence with antibodies to another disease in the studied goat herds. The results of all 30 tested herds in which antibodies against at least one of the studied pathogens were found are presented.

Number of Pathogen Antibodies	Pathogen’s Name	Number of Positive Samples	% of All Positive Results
1	*Toxoplasma gondii*	116	30.5
	*Hypoderma* spp.	1	0.54
	CAEV	54	14.2
Total		171	45
2	*Toxoplasma gondii* + *Hypoderma* spp.	6	1.57
	*Toxoplasma gondii* + *Neospora caninum*	2	0.54
	*Neospora caninum* + *Mycoplasma agalactiae*	1	0.27
	*Toxoplasma gondii* + CAEV	19	5
Total		28	7.4
3	*Toxoplasma gondii* + *Neospora caninum* + CAEV	1	0.3
Total		1	0.3
At least one		200	52.6

## Data Availability

The original contributions presented in this study are included in the article and Supplementary Material. Further inquiries can be directed to the corresponding author.

## Supplementary Materials

The following supporting information can be downloaded at: https://www.mdpi.com/article/10.3390/vetsci13010086/s1, Representative ELISA results for all pathogens are shown in Table S1: Toxoplasmosis, Table S2: BVD, Table S3: Neospora caninum, Table S4: Q fever, Table S5: Hypodermosis, Table S6: Mycoplasma agalactiae, Table S7: CAE.

## Abbreviations

The following abbreviations are used in this manuscript:

CAEV	Caprine-arthritis–encephalitis Virus
ELISA	Enzyme-Linked Immunosorbent Assay
CI	Confidence Interval
