# Supplementary material for: Serological Insights into Infectious Agents Circulating in Lithuanian Goats
Source: Vet Sci. 2026 Jan 15;13(1):86. doi: 10.3390/vetsci13010086 (PMC12846376; doi:10.3390/vetsci13010086)
Supplement: Supplementary file 1 [file vetsci-13-00086-s001.zip › Supplementary Table S1. Toxoplasmosis.pdf]

Toxoplasmosis 1 pl. 1-92

1	2	3	4	5	6	7	8	9	10	11	12
0,038	0,133	2,423	2,692	0,078	0,137	0,342	0,167	0,108	0,784	0,18	0,083
0,04	1,77	2,792	0,138	3,168	2,252	2,373	0,074	2,098	2,494	0,272	0,196
1,386	1,084	2,184	2,588	2,584	0,441	0,177	0,121	2,442	0,425	0,336	0,119
1,355	0,287	0,169	1,968	0,08	2,447	0,223	0,106	2,339	1,939	0,074	0,656
0,253	0,243	2,197	2,495	0,168	2,927	2,337	0,109	2,327	0,194	0,12	0,171
0,309	0,127	2,17	0,209	0,356	0,163	0,407	0,111	2,362	0,361	0,219	0,361
0,137	0,185	2,591	2,493	2,123	2,735	0,209	0,169	0,847	2,846	0,54	0,077
0,406	2,013	0,284	0,087	0,126	2,547	0,241	0,186	1,434	0,13	0,135	0,137

	7,06	179,05	199,25	2,93	7,36	22,76	9,61	5,18	55,95	10,59	3,30
	130,00	206,76	7,44	235,00	166,20	175,29	2,63	154,64	184,38	17,50	11,79
	78,48	161,10	191,44	191,14	30,19	10,36	6,16	180,47	28,99	22,31	6,01
	18,63	9,76	144,87	3,08	180,85	13,82	5,03	172,74	142,70	2,63	46,34
16,07	15,32	162,07	184,45	9,69	216,90	172,59	5,26	171,84	11,64	6,08	9,91
20,28	6,61	160,05	12,77	23,81	9,31	27,64	5,41	174,46	24,18	13,52	24,18
7,36	10,97	191,66	184,30	156,52	202,48	12,77	9,76	60,68	210,81	37,63	2,85
27,56	148,25	18,40	3,60	6,53	188,36	15,17	11,04	104,77	6,83	7,21	7,36

Toxoplasmosis 2 pl. 93-184

1	2	3	4	5	6	7	8	9	10	11	12
0,04	0,125	0,23	0,315	2,8	1,503	0,128	0,117	0,17	1,837	0,339	0,116
0,044	0,235	0,294	0,127	1,78	2,029	0,174	0,136	0,171	0,323	2,123	0,268
0,967	1,941	2,023	0,271	2,244	0,158	0,246	0,112	0,17	1,937	2,455	0,092
0,999	2,613	0,376	0,983	2,521	0,57	0,136	0,096	0,068	0,112	2,694	0,156
0,538	2,505	0,605	1,074	2,848	0,182	0,103	1,154	0,122	0,365	0,201	0,104
0,242	2,227	0,988	2,35	2,738	0,115	0,229	0,126	0,367	1,346	1,842	0,18
2,507	0,156	0,212	1,02	2,667	0,236	0,275	0,431	0,222	0,189	2,464	0,131
1,225	2,29	0,15	1,417	1,693	0,198	0,121	0,165	0,41	0,494	0,163	0,318

	8,82	19,98	29,01	293,09	155,26	9,14	7,97	13,60	190,75	31,56	7,86
	20,51	26,78	9,03	184,70	211,16	14,03	9,99	13,71	29,86	221,15	24,02
	201,81	210,52	24,34	234,01	12,33	21,68	7,44	13,60	201,38	256,43	5,31
	273,22	35,49	100,00	263,44	56,11	9,99	5,74	2,76	7,44	281,83	12,11
52,71	261,74	59,83	109,67	298,19	14,88	6,48	118,17	8,50	34,33	16,90	6,59
21,25	232,20	100,53	245,27	286,50	7,76	19,87	8,93	34,54	138,58	191,29	14,67
261,96	12,11	18,07	103,93	278,96	20,62	24,76	41,34	19,13	15,62	257,39	9,46
125,72	238,89	11,48	146,12	175,45	16,58	8,40	13,07	39,11	48,03	12,86	29,33

Toxoplasmosis 3 pl. 185-276

1	2	3	4	5	6	7	8	9	10	11	12
0,041	0,256	2,547	0,545	1,746	0,239	0,147	2,528	2,13	0,294	0,217	2,197
0,041	0,41	0,152	0,548	0,14	0,175	0,441	0,468	0,274	2,27	2,224	1,77
0,934	0,149	0,289	2,161	2,519	0,204	0,203	0,201	0,387	0,307	2,487	0,289
0,968	0,162	0,195	2,483	2,143	0,328	0,292	0,257	0,186	1,066	0,118	2,799
0,175	2,566	2,456	0,477	0,143	0,83	2,131	0,192	0,136	0,163	0,137	2,004
0,218	0,175	1,919	0,21	0,12	2,033	0,765	0,248	2,518	2,797	0,207	0,093
0,096	0,423	0,245	2,006	0,176	2,145	0,211	1,923	2,073	2,168	2,585	2,264
0,288	0,509	0,563	0,732	0,191	0,304	0,303	0,195	1,429	0,22	0,383	0,14

	23,63	275,38	55,38	187,36	21,76	11,65	273,30	229,56	27,80	19,34	236,92
	40,55	12,20	55,71	10,88	14,73	43,96	46,92	25,60	244,95	239,89	190,00
	11,87	27,25	232,97	272,31	17,91	17,80	17,58	38,02	29,23	268,79	27,25
	13,30	16,92	268,35	230,99	31,54	27,58	23,74	15,93	112,64	8,46	303,08
14,73	277,47	265,38	47,91	11,21	86,70	229,67	16,59	10,44	13,41	10,55	215,71
19,45	14,73	206,37	18,57	8,68	218,90	79,56	22,75	272,20	302,86	18,24	5,71
6,04	41,98	22,42	215,93	14,84	231,21	18,68	206,81	223,30	233,74	279,56	244,29
27,14	51,43	57,36	75,93	16,48	28,90	28,79	16,92	152,53	19,67	37,58	10,88

Toxoplasmosis 4 pl. 277-368

1	2	3	4	5	6	7	8	9	10	11	12
0,04	2,477	0,118	2,302	1,674	0,095	0,119	0,208	0,367	0,233	1,322	0,195
0,039	0,322	0,301	2,351	2,59	0,196	0,442	0,271	0,229	0,096	1,727	0,191
0,995	0,615	0,117	2,638	0,085	1,029	0,242	0,186	0,226	0,074	1,071	2,41
1,015	1,748	3,039	0,181	2,578	0,235	0,178	0,272	2,186	0,124	2,486	0,434
0,119	0,133	2,695	2,295	0,102	1,674	0,381	0,32	0,248	0,377	1,591	1,484
0,167	0,114	2,952	1,586	0,53	0,238	0,632	0,25	2,053	0,207	2,643	0,276
0,117	0,378	2,43	2,849	0,209	0,152	0,116	0,124	2,61	0,191	2,034	0,238
0,161	0,114	0,599	0,335	0,093	0,079	0,209	0,101	0,393	0,341	1,859	0,315

	252,46	8,13	234,33	169,29	5,75	8,23	17,45	33,92	20,04	132,83	16,11
	29,26	27,08	239,41	264,16	16,21	41,69	23,98	19,63	5,85	174,78	15,69
	59,61	8,03	269,14	4,71	102,49	20,97	15,17	19,32	3,57	106,84	245,52
	176,95	310,67	14,66	262,92	20,25	14,34	24,08	222,32	8,75	253,39	40,86
8,23	9,68	275,04	233,61	6,47	169,29	35,37	29,05	21,60	34,96	160,69	149,61
13,21	7,72	301,66	160,18	50,80	20,56	61,37	21,80	208,54	17,35	269,65	24,50
8,03	35,06	247,59	290,99	17,56	11,65	7,92	8,75	266,24	15,69	206,58	20,56
12,58	7,72	57,95	30,61	5,54	4,09	17,56	6,37	36,61	31,23	188,45	28,53
