# Supplementary material for: Serological Insights into Infectious Agents Circulating in Lithuanian Goats
Source: Vet Sci. 2026 Jan 15;13(1):86. doi: 10.3390/vetsci13010086 (PMC12846376; doi:10.3390/vetsci13010086)
Supplement: Supplementary file 1 [file vetsci-13-00086-s001.zip › Supplementary Table S2. BVD.pdf]

BVD/MD/BD virus p80-125 protein **1 plate**

	1	2	3	4	5	6	7	8	9	10	11	12
A		89,709	95,737	94,434	98,127	87,809	85,039	97,421	96,823	93,402	89,709	90,741
B		93,456	93,511	92,859	98,452	96,280	90,524	93,728	89,492	85,365	97,638	103,285
C		90,415	91,501	88,135	85,637	94,325	96,986	84,388	96,226	90,741	97,312	96,823
D		84,388	87,266	83,139	84,931	91,284	84,116	87,537	81,944	61,689	91,773	95,031
E	91,067	91,447	85,963	86,451	90,524	88,243	90,958	81,781	93,728	88,895	91,773	97,149
F	90,687	90,850	82,759	86,940	85,908	87,374	89,655	83,302	94,271	91,501	93,402	95,737
G	89,547	90,361	91,284	89,981	89,601	96,878	92,750	93,294	94,162	99,213	96,117	100,570
H	93,837	88,569	93,402	96,009	96,009	83,845	89,438	78,794	96,226	98,887	94,0538	84,714

	1	2	3	4	5	6	7	8	9	10	11	12
A	1,872	1,652	1,763	1,739	1,807	1,617	1,566	1,794	1,783	1,72	1,652	1,671
B	1,811	1,721	1,722	1,71	1,813	1,773	1,667	1,726	1,648	1,572	1,798	1,902
C	0,08	1,665	1,685	1,623	1,577	1,737	1,786	1,554	1,772	1,671	1,792	1,783
D	0,071	1,554	1,607	1,531	1,564	1,681	1,549	1,612	1,509	1,136	1,69	1,75
E	1,677	1,684	1,583	1,592	1,667	1,625	1,675	1,506	1,726	1,637	1,69	1,789
F	1,67	1,673	1,524	1,601	1,582	1,609	1,651	1,534	1,736	1,685	1,72	1,763
G	1,649	1,664	1,681	1,657	1,65	1,784	1,708	1,718	1,734	1,827	1,77	1,852
H	1,728	1,631	1,72	1,768	1,768	1,544	1,647	1,451	1,772	1,821	1,732	1,56

BVD/MD/BD virus p80-125 protein **2 plate**

	1	2	3	4	5	6	7	8	9	10	11	12
A		102,612	100,272	97,334	91,404	101,523	99,238	94,668	100,598	104,461	98,966	103,101
B		98,531	93,308	97,062	96,464	95,212	97,661	99,946	98,640	97,661	95,702	100,326
C		83,732	93,689	88,575	90,316	84,984	90,370	74,102	91,893	88,411	85,963	95,212
D		92,274	88,575	88,901	89,445	91,567	87,378	96,681	90,098	91,676	87,813	85,201
E	70,566	95,212	91,839	89,282	90,588	92,274	82,535	92,655	92,927	94,995	94,886	87,704
F	85,800	81,338	76,170	86,561	85,909	92,818	93,471	90,642	89,989	92,709	90,044	90,642
G	95,702	94,614	84,657	95,539	96,028	91,404	96,627	97,225	92,329	97,225	90,642	88,575
H	76,931	97,552	96,464	84,603	95,049	90,914	93,580	98,640	92,437	95,865	87,7584	87,922

	1	2	3	4	5	6	7	8	9	10	11	12
A	1,891	1,886	1,843	1,789	1,68	1,866	1,824	1,74	1,849	1,92	1,819	1,895
B	1,785	1,811	1,715	1,784	1,773	1,75	1,795	1,837	1,813	1,795	1,759	1,844
C	0,076	1,539	1,722	1,628	1,66	1,562	1,661	1,362	1,689	1,625	1,58	1,75
D	0,068	1,696	1,628	1,634	1,644	1,683	1,606	1,777	1,656	1,685	1,614	1,566
E	1,297	1,75	1,688	1,641	1,665	1,696	1,517	1,703	1,708	1,746	1,744	1,612
F	1,577	1,495	1,4	1,591	1,579	1,706	1,718	1,666	1,654	1,704	1,655	1,666
G	1,759	1,739	1,556	1,756	1,765	1,68	1,776	1,787	1,697	1,787	1,666	1,628
H	1,414	1,793	1,773	1,555	1,747	1,671	1,72	1,813	1,699	1,762	1,613	1,616

BVD/MD/BD virus p80-125 protein 3 plate

	1	2	3	4	5	6	7	8	9	10	11	12
A		91,119	84,377	95,044	96,101	84,730	93,686	96,201	88,201	93,585	94,491	104,302
B		79,648	81,258	84,629	86,541	91,321	88,000	77,686	85,031	90,818	92,025	99,572
C		81,157	78,289	89,409	77,182	88,956	78,189	85,132	90,767	92,579	90,365	98,214
D		79,748	84,327	86,642	77,836	78,491	75,421	85,937	80,151	91,119	81,761	92,780
E	83,472	84,226	81,459	89,208	89,107	85,686	83,824	86,692	88,101	89,358	93,937	70,491
F	80,252	81,258	87,698	89,308	85,082	80,252	72,352	84,377	83,522	96,101	90,063	90,868
G	86,088	77,535	83,572	78,591	92,528	93,786	87,044	83,774	95,094	91,220	93,384	88,906
H	75,623	79,799	100,075	87,145	93,082	93,736	96,453	99,522	92,528	94,138	94,2893	97,509

	1	2	3	4	5	6	7	8	9	10	11	12
A	2,038	1,811	1,677	1,889	1,91	1,684	1,862	1,912	1,753	1,86	1,878	2,073
B	1,937	1,583	1,615	1,682	1,72	1,815	1,749	1,544	1,69	1,805	1,829	1,979
C	0,083	1,613	1,556	1,777	1,534	1,768	1,554	1,692	1,804	1,84	1,796	1,952
D	0,08	1,585	1,676	1,722	1,547	1,56	1,499	1,708	1,593	1,811	1,625	1,844
E	1,659	1,674	1,619	1,773	1,771	1,703	1,666	1,723	1,751	1,776	1,867	1,401
F	1,595	1,615	1,743	1,775	1,691	1,595	1,438	1,677	1,66	1,91	1,79	1,806
G	1,711	1,541	1,661	1,562	1,839	1,864	1,73	1,665	1,89	1,813	1,856	1,767
H	1,503	1,586	1,989	1,732	1,85	1,863	1,917	1,978	1,839	1,871	1,874	1,938

BVD/MD/BD virus p80-125 protein 4 plate

	1	2	3	4	5	6	7	8	9	10	11	12
A		96,948	101,609	100,888	93,063	98,613	97,225	92,841	92,453	86,959	101,998	90,122
B		91,398	95,893	86,016	97,114	96,781	100,166	93,951	97,725	93,452	97,780	103,441
C		80,189	92,453	93,008	93,341	95,228	94,284	97,281	83,130	90,178	85,461	89,623
D		82,575	87,569	82,131	84,129	85,794	94,784	86,016	76,859	87,625	81,687	96,504
E	75,139	89,567	87,625	85,405	91,065	90,511	97,447	91,787	74,917	86,016	92,453	82,297
F	86,071	91,343	92,730	85,960	91,953	97,503	95,450	92,841	90,011	89,290	94,007	91,898
G	82,464	90,844	94,562	95,394	93,452	89,512	88,457	86,626	89,900	92,508	99,001	94,062
H	85,350	99,501	103,496	97,891	95,228	101,942	98,446	95,949	99,889	85,849	82,7414	99,112

	1	2	3	4	5	6	7	8	9	10	11	12
A	1,815	1,747	1,831	1,818	1,677	1,777	1,752	1,673	1,666	1,567	1,838	1,624
B	1,789	1,647	1,728	1,55	1,75	1,744	1,805	1,693	1,761	1,684	1,762	1,864
C	0,089	1,445	1,666	1,676	1,682	1,716	1,699	1,753	1,498	1,625	1,54	1,615
D	0,081	1,488	1,578	1,48	1,516	1,546	1,708	1,55	1,385	1,579	1,472	1,739
E	1,354	1,614	1,579	1,539	1,641	1,631	1,756	1,654	1,35	1,55	1,666	1,483
F	1,551	1,646	1,671	1,549	1,657	1,757	1,72	1,673	1,622	1,609	1,694	1,656
G	1,486	1,637	1,704	1,719	1,684	1,613	1,594	1,561	1,62	1,667	1,784	1,695
H	1,538	1,793	1,865	1,764	1,716	1,837	1,774	1,729	1,8	1,547	1,491	1,786
