# Supplementary material for: Serological Insights into Infectious Agents Circulating in Lithuanian Goats
Source: Vet Sci. 2026 Jan 15;13(1):86. doi: 10.3390/vetsci13010086 (PMC12846376; doi:10.3390/vetsci13010086)
Supplement: Supplementary file 1 [file vetsci-13-00086-s001.zip › Supplementary Table S3. Neospora caninum.pdf]

**Neospora caninum 1 pl.**

| | 1 | 2 | 3 | 4 | 5 | 6 | 7 | 8 | 9 | 10 | 11 | 12 |
|---|---|---|---|---|---|---|---|---|---|---|---|---|
| A | 0,04 | 0,061 | 0,051 | 0,079 | 0,068 | 0,172 | 0,073 | 0,131 | 0,054 | 0,062 | 0,071 | 0,147 |
| B | 0,04 | 0,061 | 0,279 | 0,189 | 0,063 | 0,092 | 0,214 | 0,065 | 0,088 | 0,093 | 0,051 | 0,1 |
| C | 1,167 | 0,116 | 0,078 | 0,075 | 0,099 | 0,092 | 0,091 | 0,101 | 0,152 | 0,102 | 0,266 | 0,095 |
| D | 1,16 | 0,054 | 0,078 | 1,083 | 0,051 | 0,087 | 0,125 | 0,069 | 0,329 | 0,172 | 0,063 | 0,224 |
| E | 0,058 | 0,056 | 0,095 | 0,072 | 0,077 | 0,062 | 0,085 | 0,107 | 0,092 | 0,082 | 0,059 | 0,12 |
| F | 0,092 | 0,1 | 0,135 | 0,075 | 0,092 | 0,117 | 0,097 | 0,077 | 0,162 | 0,095 | 0,1 | 0,149 |
| G | 0,049 | 0,084 | 0,194 | 0,157 | 0,328 | 0,111 | 0,158 | 0,159 | 0,089 | 0,064 | 0,184 | 0,187 |
| H | 0,146 | 0,069 | 0,061 | 0,073 | 0,061 | 0,14 | 0,061 | 0,207 | 0,075 | 0,231 | 0,136 | 0,12 |

| | 1 | 2 | 3 | 4 | 5 | 6 | 7 | 8 | 9 | 10 | 11 | 12 |
|---|---|---|---|---|---|---|---|---|---|---|---|---|
| A | | 1,87 | 0,98 | 3,47 | 2,49 | 11,75 | 2,94 | 8,10 | 1,25 | 1,96 | 2,76 | 9,52 |
| B | | 1,87 | 21,27 | 13,26 | 2,05 | 4,63 | 15,49 | 2,23 | 4,27 | 4,72 | 0,98 | 5,34 |
| C | | 6,76 | 3,38 | 3,12 | 5,25 | 4,63 | 4,54 | 5,43 | 9,97 | 5,52 | 20,12 | 4,90 |
| D | | 1,25 | 3,38 | 92,83 | 0,98 | 4,18 | 7,57 | 2,58 | 25,72 | 11,75 | 2,05 | 16,38 |
| E | 1,60 | 1,42 | 4,90 | 2,85 | 3,29 | 1,96 | 4,01 | 5,96 | 4,63 | 3,74 | 1,69 | 7,12 |
| F | 4,63 | 5,34 | 8,46 | 3,12 | 4,63 | 6,85 | 5,07 | 3,29 | 10,86 | 4,90 | 5,34 | 9,70 |
| G | 0,80 | 3,92 | 13,71 | 10,41 | 25,63 | 6,32 | 10,50 | 10,59 | 4,36 | 2,14 | 12,82 | 13,08 |
| H | 9,43 | 2,58 | 1,87 | 2,94 | 1,87 | 8,90 | 1,87 | 14,86 | 3,12 | 17,00 | 8,54 | 7,12 |

**Neospora caninum 2 pl.**

| | 1 | 2 | 3 | 4 | 5 | 6 | 7 | 8 | 9 | 10 | 11 | 12 |
|---|---|---|---|---|---|---|---|---|---|---|---|---|
| A | 0,039 | 0,204 | 0,163 | 0,12 | 0,128 | 0,146 | 0,237 | 0,247 | 0,134 | 0,173 | 0,096 | 0,063 |
| B | 0,038 | 0,193 | 0,17 | 0,051 | 0,258 | 0,073 | 0,125 | 0,189 | 0,102 | 0,254 | 0,158 | 0,079 |
| C | 1,177 | 0,177 | 0,153 | 0,053 | 0,235 | 0,202 | 0,088 | 0,079 | 0,201 | 0,403 | 0,121 | 0,135 |
| D | 1,198 | 0,064 | 0,153 | 0,066 | 0,046 | 0,071 | 0,198 | 0,076 | 0,054 | 0,055 | 0,11 | 0,086 |
| E | 0,2 | 0,195 | 0,064 | 0,067 | 0,132 | 0,202 | 0,199 | 0,161 | 0,087 | 0,107 | 0,156 | 0,125 |
| F | 0,116 | 0,091 | 0,472 | 0,048 | 0,313 | 0,203 | 0,074 | 0,083 | 0,161 | 0,102 | 0,114 | 0,217 |
| G | 0,077 | 0,099 | 0,048 | 0,121 | 0,068 | 0,174 | 0,168 | 0,093 | 0,136 | 0,102 | 0,063 | 0,075 |
| H | 0,047 | 0,08 | 0,129 | 0,069 | 0,125 | 0,174 | 0,397 | 0,078 | 0,065 | 0,222 | 0,078 | 0,184 |

| | 1 | 2 | 3 | 4 | 5 | 6 | 7 | 8 | 9 | 10 | 11 | 12 |
|---|---|---|---|---|---|---|---|---|---|---|---|---|
| A | | 14,40 | 10,84 | 7,09 | 7,79 | 9,36 | 17,28 | 18,15 | 8,31 | 11,71 | 5,00 | 2,13 |
| B | | 13,45 | 11,44 | 1,09 | 19,10 | 3,00 | 7,53 | 13,10 | 5,53 | 18,76 | 10,40 | 3,52 |
| C | | 12,05 | 9,97 | 1,26 | 17,10 | 14,23 | 4,31 | 3,52 | 14,14 | 31,72 | 7,18 | 8,40 |
| D | | 2,22 | 9,97 | 2,39 | 0,65 | 2,83 | 13,88 | 3,26 | 1,35 | 1,44 | 6,22 | 4,13 |
| E | 14,06 | 13,62 | 2,22 | 2,48 | 8,14 | 14,23 | 13,97 | 10,66 | 4,22 | 5,96 | 10,23 | 7,53 |
| F | 6,74 | 4,57 | 37,73 | 0,83 | 23,89 | 14,32 | 3,09 | 3,87 | 10,66 | 5,53 | 6,57 | 15,54 |
| G | 3,35 | 5,27 | 0,83 | 7,18 | 2,57 | 11,79 | 11,27 | 4,74 | 8,49 | 5,53 | 2,13 | 3,18 |
| H | 0,74 | 3,61 | 7,88 | 2,65 | 7,53 | 11,79 | 31,20 | 3,44 | 2,31 | 15,97 | 3,44 | 12,66 |

**Neospora caninum 3 pl.**

| | 1 | 2 | 3 | 4 | 5 | 6 | 7 | 8 | 9 | 10 | 11 | 12 |
|---|---|---|---|---|---|---|---|---|---|---|---|---|
| A | 0,042 | 0,064 | 0,086 | 0,052 | 0,066 | 0,096 | 0,126 | 0,22 | 0,106 | 0,2 | 0,442 | 0,065 |
| B | 0,043 | 0,066 | 0,102 | 0,24 | 0,098 | 0,108 | 0,085 | 0,083 | 0,098 | 0,13 | 0,082 | 0,159 |
| C | 1,167 | 0,179 | 0,229 | 0,115 | 0,134 | 0,154 | 0,133 | 0,123 | 0,106 | 0,106 | 0,113 | 0,096 |
| D | 1,132 | 0,07 | 0,094 | 0,202 | 0,09 | 0,094 | 0,124 | 0,085 | 0,076 | 0,071 | 0,082 | 0,093 |
| E | 0,072 | 0,189 | 0,101 | 0,071 | 0,074 | 0,115 | 0,144 | 0,13 | 0,111 | 0,152 | 0,128 | 0,094 |
| F | 0,158 | 0,093 | 0,119 | 0,057 | 0,143 | 0,146 | 0,12 | 0,135 | 0,438 | 0,129 | 0,129 | 0,078 |
| G | 0,062 | 0,172 | 0,082 | 0,177 | 0,088 | 0,107 | 0,134 | 0,086 | 0,108 | 0,214 | 0,074 | 0,081 |
| H | 0,285 | 0,104 | 0,092 | 0,132 | 0,09 | 0,154 | 0,131 | 0,195 | 0,116 | 0,07 | 0,069 | 0,06 |

| | 1 | 2 | 3 | 4 | 5 | 6 | 7 | 8 | 9 | 10 | 11 | 12 |
|---|---|---|---|---|---|---|---|---|---|---|---|---|
| A | | 1,94 | 3,93 | 0,86 | 2,12 | 4,83 | 7,54 | 16,03 | 5,74 | 14,23 | 36,09 | 2,03 |
| B | | 2,12 | 5,37 | 17,84 | 5,01 | 5,92 | 3,84 | 3,66 | 5,01 | 7,90 | 3,57 | 10,52 |
| C | | 12,33 | 16,85 | 6,55 | 8,27 | 10,07 | 8,18 | 7,27 | 5,74 | 5,74 | 6,37 | 4,83 |
| D | | 2,48 | 4,65 | 14,41 | 4,29 | 4,65 | 7,36 | 3,84 | 3,03 | 2,57 | 3,57 | 4,56 |
| E | 2,66 | 13,23 | 5,28 | 2,57 | 2,85 | 6,55 | 9,17 | 7,90 | 6,19 | 9,89 | 7,72 | 4,65 |
| F | 10,43 | 4,56 | 6,91 | 1,31 | 9,08 | 9,35 | 7,00 | 8,36 | 35,73 | 7,81 | 7,81 | 3,21 |
| G | 1,76 | 11,70 | 3,57 | 12,15 | 4,11 | 5,83 | 8,27 | 3,93 | 5,92 | 15,49 | 2,85 | 3,48 |
| H | 21,91 | 5,56 | 4,47 | 8,08 | 4,29 | 10,07 | 7,99 | 13,78 | 6,64 | 2,48 | 2,39 | 1,58 |

**Neospora caninum 4 pl.**

| | 1 | 2 | 3 | 4 | 5 | 6 | 7 | 8 | 9 | 10 | 11 | 12 |
|---|---|---|---|---|---|---|---|---|---|---|---|---|
| A | 0,038 | 0,081 | 0,09 | 0,067 | 0,111 | 0,284 | 0,164 | 0,2 | 0,098 | 0,305 | 0,072 | 0,087 |
| B | 0,04 | 0,061 | 0,1 | 0,153 | 0,096 | 0,171 | 0,083 | 0,114 | 0,121 | 0,067 | 0,528 | 0,121 |
| C | 0,931 | 0,067 | 0,145 | 0,056 | 0,056 | 0,091 | 0,122 | 0,081 | 0,116 | 0,091 | 0,07 | 0,062 |
| D | 0,92 | 0,071 | 0,46 | 0,187 | 0,134 | 0,088 | 0,089 | 0,106 | 0,076 | 0,137 | 0,093 | 0,11 |
| E | 0,071 | 0,082 | 0,076 | 0,258 | 0,208 | 0,064 | 0,24 | 0,046 | 0,091 | 0,15 | 0,099 | 1,024 |
| F | 0,125 | 0,158 | 0,063 | 0,265 | 0,091 | 0,146 | 0,241 | 0,074 | 0,084 | 0,073 | 0,254 | 0,081 |
| G | 0,523 | 0,103 | 0,115 | 0,131 | 0,086 | 0,063 | 0,188 | 0,171 | 0,233 | 0,112 | 0,084 | 0,067 |
| H | 0,09 | 0,096 | 0,061 | 0,15 | 0,074 | 0,086 | 0,093 | 0,1 | 0,136 | 0,115 | 0,14 | 0,08 |

| | 1 | 2 | 3 | 4 | 5 | 6 | 7 | 8 | 9 | 10 | 11 | 12 |
|---|---|---|---|---|---|---|---|---|---|---|---|---|
| A | | 4,74 | 5,75 | 3,16 | 8,12 | 27,64 | 14,10 | 18,16 | 6,66 | 30,01 | 3,72 | 5,41 |
| B | | 2,48 | 6,88 | 12,86 | 6,43 | 14,89 | 4,96 | 8,46 | 9,25 | 3,16 | 55,16 | 9,25 |
| C | | 3,16 | 11,96 | 1,92 | 1,92 | 5,87 | 9,36 | 4,74 | 8,69 | 5,87 | 3,50 | 2,59 |
| D | | 3,61 | 47,49 | 16,69 | 10,72 | 5,53 | 5,64 | 7,56 | 4,17 | 11,05 | 6,09 | 8,01 |
| E | 3,61 | 4,85 | 4,17 | 24,70 | 19,06 | 2,82 | 22,67 | 0,79 | 5,87 | 12,52 | 6,77 | 111,11 |
| F | 9,70 | 13,42 | 2,71 | 25,49 | 5,87 | 12,07 | 22,79 | 3,95 | 5,08 | 3,84 | 24,25 | 4,74 |
| G | 54,60 | 7,22 | 8,57 | 10,38 | 5,30 | 2,71 | 16,81 | 14,89 | 21,88 | 8,23 | 5,08 | 3,16 |
| H | 5,75 | 6,43 | 2,48 | 12,52 | 3,95 | 5,30 | 6,09 | 6,88 | 10,94 | 8,57 | 11,39 | 4,62 |
