# Supplementary material for: Serological Insights into Infectious Agents Circulating in Lithuanian Goats
Source: Vet Sci. 2026 Jan 15;13(1):86. doi: 10.3390/vetsci13010086 (PMC12846376; doi:10.3390/vetsci13010086)
Supplement: Supplementary file 1 [file vetsci-13-00086-s001.zip › Supplementary Table S4. Q fever.pdf]

Q FEWER 1 pl.

1	2	3	4	5	6	7	8	9	10	11	12
0,039	0,074	0,056	0,087	0,077	0,101	0,088	0,142	0,061	0,07	0,072	0,071
0,042	0,062	0,678	0,083	0,094	0,066	0,097	0,075	0,074	0,131	0,069	0,121
1,14	0,145	0,07	0,132	0,055	0,063	0,115	0,097	0,145	0,084	0,099	0,074
1,14	0,052	0,08	0,073	0,049	0,07	0,136	0,068	0,088	0,071	0,076	0,097
0,088	0,051	0,088	0,063	0,076	0,084	0,082	0,408	0,072	0,078	0,17	0,069
0,098	0,053	0,067	0,077	0,079	0,089	0,081	0,061	0,091	0,062	0,123	0,071
0,07	0,056	0,077	0,055	0,051	0,059	0,063	0,091	0,08	0,076	0,1	0,06
0,074	0,057	0,061	0,059	0,126	0,085	0,067	0,072	0,081	0,159	0,113	0,06

	3,05	1,41	4,23	3,32	5,50	4,32	9,23	1,86	2,68	2,86	2,77
	1,96	57,98	3,87	4,87	2,32	5,14	3,14	3,05	8,23	2,59	7,32
	9,50	2,68	8,32	1,32	2,05	6,78	5,14	9,50	3,96	5,32	3,05
	1,05	3,59	2,96	0,77	2,68	8,69	2,50	4,32	2,77	3,23	5,14
4,32	0,95	4,32	2,05	3,23	3,96	3,77	33,42	2,86	3,41	11,78	2,59
5,23	1,14	2,41	3,32	3,50	4,41	3,68	1,86	4,59	1,96	7,50	2,77
2,68	1,41	3,32	1,32	0,95	1,68	2,05	4,59	3,59	3,23	5,41	1,77
3,05	1,50	1,86	1,68	7,78	4,05	2,41	2,86	3,68	10,78	6,59	1,77

Q FEWER 2 pl.

1	2	3	4	5	6	7	8	9	10	11	12
0,042	0,054	0,065	0,06	0,077	0,067	0,058	0,064	0,075	0,066	0,095	0,061
0,044	0,054	0,055	0,111	0,062	0,104	0,069	0,061	0,073	0,071	0,066	0,13
1,102	0,077	0,059	0,092	0,092	0,055	0,071	0,058	0,068	0,061	0,057	0,096
1,069	0,055	0,224	0,067	0,055	0,104	0,077	0,056	0,109	0,071	0,09	0,09
0,057	0,084	0,057	0,057	0,075	0,061	0,064	0,336	0,085	0,074	0,096	0,071
0,065	0,068	0,076	0,049	0,083	0,075	0,106	0,051	0,081	0,079	0,086	0,09
0,058	0,051	0,064	0,066	0,064	0,1	0,066	0,065	0,094	0,064	0,085	0,106
0,037	0,056	0,08	0,07	0,06	0,074	0,058	0,057	0,131	0,052	0,101	0,111

	1,06	2,11	1,63	3,26	2,30	1,44	2,01	3,07	2,21	4,99	1,73
	1,06	1,15	6,52	1,82	5,85	2,49	1,73	2,88	2,69	2,21	8,35
	3,26	1,53	4,70	4,70	1,15	2,69	1,44	2,40	1,73	1,34	5,08
	1,15	17,36	2,30	1,15	5,85	3,26	1,25	6,33	2,69	4,51	4,51
1,34	3,93	1,34	1,34	3,07	1,73	2,01	28,11	4,03	2,97	5,08	2,69
2,11	2,40	3,17	0,58	3,84	3,07	6,04	0,77	3,65	3,45	4,12	4,51
1,44	0,77	2,01	2,21	2,01	5,47	2,21	2,11	4,89	2,01	4,03	6,04
-0,58	1,25	3,55	2,59	1,63	2,97	1,44	1,34	8,44	0,86	5,56	6,52

Q Fever 3 pl.

1	2	3	4	5	6	7	8	9	10	11	12
0,039	0,064	0,069	0,159	0,086	0,062	0,054	0,055	0,119	0,062	0,066	0,112
0,042	0,073	0,072	0,055	0,29	0,057	0,063	0,063	0,092	0,1	0,106	0,071
1,052	0,063	0,107	0,084	0,08	0,047	0,075	0,063	0,095	0,058	0,062	0,063
1,056	0,092	0,058	0,124	0,064	0,357	0,071	0,058	0,065	0,065	0,061	0,061
0,068	0,061	0,06	0,089	0,052	0,053	0,079	0,057	0,07	0,063	0,058	0,281
0,07	0,058	0,076	0,076	0,073	0,065	0,048	0,057	0,061	0,059	0,075	0,059
0,063	0,091	0,066	0,093	0,057	0,062	0,058	0,054	0,085	0,049	0,052	0,057
0,174	0,066	0,059	0,391	0,06	0,068	0,071	0,061	0,135	0,052	0,063	0,063

	2,32	2,81	11,69	4,49	2,12	1,33	1,43	7,75	2,12	2,52	7,05
	3,21	3,11	1,43	24,62	1,63	2,22	2,22	5,08	5,87	6,46	3,01
	2,22	6,56	4,29	3,90	0,64	3,40	2,22	5,38	1,73	2,12	2,22
	5,08	1,73	8,24	2,32	31,23	3,01	1,73	2,42	2,42	2,02	2,02
2,71	2,02	1,92	4,79	1,13	1,23	3,80	1,63	2,91	2,22	1,73	23,73
2,91	1,73	3,50	3,50	3,21	2,42	0,74	1,63	2,02	1,83	3,40	1,83
2,22	4,98	2,52	5,18	1,63	2,12	1,73	1,33	4,39	0,84	1,13	1,63
13,17	2,52	1,83	34,58	1,92	2,71	3,01	2,02	9,32	1,13	2,22	2,22

Q Fever 4 pl.

1	2	3	4	5	6	7	8	9	10	11	12
0,04	0,055	0,06	0,054	0,058	0,076	0,088	0,064	0,076	0,1	0,145	0,089
0,041	0,063	0,064	0,074	0,06	0,14	0,073	0,058	0,083	0,095	0,096	0,103
1,187	0,137	0,065	0,056	0,092	0,077	0,065	0,09	0,087	0,075	0,084	0,106
1,155	0,084	0,06	0,078	0,148	0,068	0,109	0,063	0,07	0,127	0,079	0,073
0,073	0,058	0,054	0,066	0,056	0,075	0,092	0,06	0,078	0,142	0,068	0,081
0,124	0,12	0,065	0,068	0,067	0,076	0,078	0,068	0,068	0,09	0,061	0,069
0,087	0,067	0,067	0,152	0,081	0,087	0,065	0,058	0,172	0,076	0,154	0,08
0,058	0,081	0,088	0,086	0,064	0,053	0,131	0,076	0,101	0,054	0,07	0,072

	1,28	1,72	1,19	1,55	3,14	4,20	2,08	3,14	5,26	9,24	4,29
	1,99	2,08	2,96	1,72	8,80	2,87	1,55	3,76	4,82	4,91	5,53
	8,54	2,17	1,37	4,56	3,23	2,17	4,38	4,11	3,05	3,85	5,79
	3,85	1,72	3,32	9,51	2,43	6,06	1,99	2,61	7,65	3,41	2,87
2,87	1,55	1,19	2,26	1,37	3,05	4,56	1,72	3,32	8,98	2,43	3,58
7,39	7,03	2,17	2,43	2,34	3,14	3,32	2,43	2,43	4,38	1,81	2,52
4,11	2,34	2,34	9,86	3,58	4,11	2,17	1,55	11,63	3,14	10,04	3,49
1,55	3,58	4,20	4,02	2,08	1,11	8,01	3,14	5,35	1,19	2,61	2,79
