# Supplementary material for: Serological Insights into Infectious Agents Circulating in Lithuanian Goats
Source: Vet Sci. 2026 Jan 15;13(1):86. doi: 10.3390/vetsci13010086 (PMC12846376; doi:10.3390/vetsci13010086)
Supplement: Supplementary file 1 [file vetsci-13-00086-s001.zip › Supplementary Table S5. Hypodermosis.pdf]

Hypodermosis
1-92

	1	2	3	4	5	6	7	8	9	10	11	12
A	0,04	0,059	0,063	0,065	0,05	0,061	0,068	0,064	0,056	0,141	0,065	0,066
B	0,04	0,055	0,497	0,181	0,064	0,06	1,439	0,05	0,156	0,13	0,063	0,094
C	0,798	0,116	0,101	0,066	0,085	0,058	0,089	0,064	0,09	0,425	0,065	0,057
D	0,832	0,054	0,14	0,059	0,055	0,07	0,079	0,056	0,091	0,102	0,065	0,085
E	0,048	0,055	0,062	0,11	0,054	0,129	2,24	0,067	0,709	0,071	0,092	0,063
F	0,079	0,085	0,07	0,066	0,061	0,201	0,075	0,064	2,674	0,064	0,105	0,226
G	0,071	0,068	0,069	0,054	0,077	0,072	0,054	0,071	0,065	0,066	0,099	0,057
H	0,057	0,069	0,42	0,052	0,063	0,212	0,061	0,072	0,247	0,075	0,103	0,059

	1	2	3	4	5	6	7	8	9	10	11	12
A		2,45	2,97	3,23	1,29	2,71	3,61	3,10	2,06	13,03	3,23	3,35
B		1,94	58,97	18,19	3,10	2,58	180,52	1,29	14,97	11,61	2,97	6,97
C		9,81	7,87	3,35	5,81	2,32	6,32	3,10	6,45	49,68	3,23	2,19
D		1,81	12,90	2,45	1,94	3,87	5,03	2,06	6,58	8,00	3,23	5,81
E	1,03	1,94	2,84	9,03	1,81	11,48	283,87	3,48	86,32	4,00	6,71	2,97
F	5,03	5,81	3,87	3,35	2,71	20,77	4,52	3,10	339,87	3,10	8,39	24,00
G	4,00	3,61	3,74	1,81	4,77	4,13	1,81	4,00	3,23	3,35	7,61	2,19
H	2,19	3,74	49,03	1,55	2,97	22,19	2,71	4,13	26,71	4,52	8,13	2,45

Hypodermosis
93-184

	1	2	3	4	5	6	7	8	9	10	11	12
A	0,041	0,064	0,067	0,059	0,074	0,049	0,069	0,092	0,237	0,562	0,069	0,067
B	0,044	0,052	0,058	0,072	0,053	0,063	0,106	0,058	0,064	0,056	0,141	0,117
C	0,888	0,066	0,059	0,084	0,078	0,067	0,116	1,754	0,092	0,089	0,066	0,081
D	0,968	0,074	0,054	0,208	0,06	0,062	0,075	0,411	0,057	0,118	0,314	0,091
E	0,075	0,061	0,081	0,057	0,06	0,177	0,077	0,117	0,081	0,092	0,107	0,081
F	0,085	0,056	0,071	0,055	0,073	0,083	0,059	0,083	0,066	0,11	0,169	0,074
G	0,088	0,057	0,056	0,084	0,065	0,078	0,098	0,083	0,067	0,135	0,095	0,069
H	0,079	0,11	0,084	0,094	0,092	0,075	0,084	0,05	0,091	0,072	0,079	0,064

	1	2	3	4	5	6	7	8	9	10	11	12
A		2,43	2,77	1,86	3,56	0,73	2,99	5,59	21,96	58,67	2,99	2,77
B		1,07	1,75	3,33	1,19	2,32	7,17	1,75	2,43	1,52	11,12	8,41
C		2,65	1,86	4,69	4,01	2,77	8,30	193,28	5,59	5,25	2,65	4,35
D		3,56	1,30	18,69	1,98	2,20	3,67	41,61	1,64	8,53	30,66	5,48
E	3,67	2,09	4,35	1,64	1,98	15,19	3,90	8,41	4,35	5,59	7,28	4,35
F	4,80	1,52	3,22	1,41	3,44	4,57	1,86	4,57	2,65	7,62	14,29	3,56
G	5,14	1,64	1,52	4,69	2,54	4,01	6,27	4,57	2,77	10,45	5,93	2,99
H	4,12	7,62	4,69	5,82	5,59	3,67	4,69	0,85	5,48	3,33	4,12	2,43
