# Supplementary material for: Serological Insights into Infectious Agents Circulating in Lithuanian Goats
Source: Vet Sci. 2026 Jan 15;13(1):86. doi: 10.3390/vetsci13010086 (PMC12846376; doi:10.3390/vetsci13010086)
Supplement: Supplementary file 1 [file vetsci-13-00086-s001.zip › Supplementary Table S6. Mycoplasma agalactiae.pdf]

Mycoplasma agalactiae 1 pl. **1-92**

	1	2	3	4	5	6	7	8	9	10	11	12
A	0,039	0,068	0,052	0,047	0,042	0,048	0,169	0,042	0,042	0,047	0,047	0,053
B	0,038	0,053	0,187	0,058	0,046	0,05	0,052	0,042	0,055	0,065	0,048	0,054
C	1,309	0,067	0,059	0,05	0,05	0,053	0,232	0,05	0,051	0,069	0,06	0,061
D	1,351	0,064	0,051	0,045	0,042	0,05	0,048	0,044	0,056	0,076	0,049	0,062
E	0,064	0,06	0,078	0,06	0,051	0,079	0,048	0,045	0,048	0,059	0,052	0,055
F	0,057	0,048	0,051	0,055	0,051	0,056	0,058	0,051	0,051	0,046	0,069	0,062
G	0,057	0,054	0,051	0,049	0,046	0,07	0,055	0,051	0,046	0,066	0,056	0,049
H	0,075	0,044	0,057	0,043	0,049	0,063	0,055	0,05	0,059	0,048	0,062	0,058

	1	2	3	4	5	6	7	8	9	10	11	12
A		2,28	1,05	0,66	0,27	0,74	10,10	0,27	0,27	0,66	0,66	1,12
B		1,12	11,50	1,51	0,58	0,89	1,05	0,27	1,28	2,05	0,74	1,20
C		2,21	1,59	0,89	0,89	1,12	14,98	0,89	0,97	2,36	1,66	1,74
D		1,97	0,97	0,50	0,27	0,89	0,74	0,43	1,36	2,90	0,81	1,82
E	1,97	1,66	3,06	1,66	0,97	3,14	0,74	0,50	0,74	1,59	1,05	1,28
F	1,43	0,74	0,97	1,28	0,97	1,36	1,51	0,97	0,97	0,58	2,36	1,82
G	1,43	1,20	0,97	0,81	0,58	2,44	1,28	0,97	0,58	2,13	1,36	0,81
H	2,83	0,43	1,43	0,35	0,81	1,90	1,28	0,89	1,59	0,74	1,82	1,51

Mycoplasma agalactiae 2 pl. **93-184**

	1	2	3	4	5	6	7	8	9	10	11	12
A	0,04	0,056	0,06	0,086	0,046	0,047	0,052	0,05	0,054	0,049	0,048	0,049
B	0,041	0,043	0,044	0,052	0,05	0,068	0,058	0,048	0,045	0,058	0,058	0,064
C	1,261	0,071	0,047	0,051	0,084	0,056	0,06	0,049	0,052	0,052	0,051	0,053
D	1,338	0,045	0,048	0,101	0,052	0,064	0,06	0,044	0,045	0,051	0,081	0,058
E	0,053	0,054	0,051	0,05	0,05	0,064	0,07	0,586	0,154	0,053	0,059	0,052
F	0,052	0,055	0,052	0,051	0,061	0,056	0,054	0,045	0,054	0,057	0,066	0,05
G	0,07	0,046	0,048	0,044	0,055	0,053	0,062	0,049	0,059	0,053	0,053	0,049
H	0,047	0,05	0,061	0,05	0,074	0,06	0,061	0,048	0,051	0,101	0,057	0,058

	1	2	3	4	5	6	7	8	9	10	11	12
A		1,23	1,55	3,61	0,44	0,52	0,91	0,75	1,07	0,68	0,60	0,68
B		0,20	0,28	0,91	0,75	2,18	1,39	0,60	0,36	1,39	1,39	1,87
C		2,42	0,52	0,83	3,46	1,23	1,55	0,68	0,91	0,91	0,83	0,99
D		0,36	0,60	4,81	0,91	1,87	1,55	0,28	0,36	0,83	3,22	1,39
E	0,99	1,07	0,83	0,75	0,75	1,87	2,34	43,33	9,02	0,99	1,47	0,91
F	0,91	1,15	0,91	0,83	1,63	1,23	1,07	0,36	1,07	1,31	2,03	0,75
G	2,34	0,44	0,60	0,28	1,15	0,99	1,71	0,68	1,47	0,99	0,99	0,68
H	0,52	0,75	1,63	0,75	2,66	1,55	1,63	0,60	0,83	4,81	1,31	1,39

Mycoplasma agalactiae 3 pl. 185-276

	1	2	3	4	5	6	7	8	9	10	11	12
A	0,044	0,06	0,058	0,044	0,062	0,051	0,064	0,047	0,07	0,114	0,057	0,049
B	0,041	0,054	0,05	0,052	0,056	0,044	0,047	0,055	0,051	0,045	0,061	0,045
C	1,12	0,045	0,069	0,066	0,05	0,046	0,047	0,049	0,051	0,048	0,056	0,049
D	1,213	0,051	0,061	0,049	0,048	0,042	0,046	0,042	0,053	0,043	0,051	0,045
E	0,05	0,051	0,043	0,043	0,042	0,041	0,053	0,07	0,051	0,039	0,121	0,056
F	0,052	0,046	0,059	0,046	0,084	0,044	0,042	0,051	0,04	0,043	0,045	0,05
G	0,072	0,105	0,051	0,059	0,043	0,05	0,043	0,045	0,049	0,053	0,044	0,082
H	0,049	0,061	0,049	0,048	0,05	0,049	0,06	0,045	0,047	0,045	0,046	0,052

	1	2	3	4	5	6	7	8	9	10	11	12
A		1,56	1,38	0,13	1,73	0,76	1,91	0,40	2,45	6,36	1,29	0,58
B		1,02	0,67	0,85	1,20	0,13	0,40	1,11	0,76	0,22	1,65	0,22
C		0,22	2,36	2,09	0,67	0,31	0,40	0,58	0,76	0,49	1,20	0,58
D		0,76	1,65	0,58	0,49	-0,04	0,31	-0,04	0,93	0,04	0,76	0,22
E	0,67	0,76	0,04	0,04	-0,04	-0,13	0,93	2,45	0,76	-0,31	6,98	1,20
F	0,85	0,31	1,47	0,31	3,69	0,13	-0,04	0,76	-0,22	0,04	0,22	0,67
G	2,62	5,56	0,76	1,47	0,04	0,67	0,04	0,22	0,58	0,93	0,13	3,51
H	0,58	1,65	0,58	0,49	0,67	0,58	1,56	0,22	0,40	0,22	0,31	0,85

Mycoplasma agalactiae 4 pl. 277-368

	1	2	3	4	5	6	7	8	9	10	11	12
A	0,039	0,068	0,052	0,047	0,042	0,048	0,169	0,042	0,042	0,047	0,047	0,053
B	0,038	0,053	0,187	0,058	0,046	0,05	0,052	0,042	0,055	0,065	0,048	0,054
C	1,309	0,067	0,059	0,05	0,05	0,053	0,232	0,05	0,051	0,069	0,06	0,061
D	1,351	0,064	0,051	0,045	0,042	0,05	0,048	0,044	0,056	0,076	0,049	0,062
E	0,064	0,06	0,078	0,06	0,051	0,079	0,048	0,045	0,048	0,059	0,052	0,055
F	0,057	0,048	0,051	0,055	0,051	0,056	0,058	0,051	0,051	0,046	0,069	0,062
G	0,057	0,054	0,051	0,049	0,046	0,07	0,055	0,051	0,046	0,066	0,056	0,049
H	0,075	0,044	0,057	0,043	0,049	0,063	0,055	0,05	0,059	0,048	0,062	0,058

	1	2	3	4	5	6	7	8	9	10	11	12
A		2,28	1,05	0,66	0,27	0,74	10,10	0,27	0,27	0,66	0,66	1,12
B		1,12	11,50	1,51	0,58	0,89	1,05	0,27	1,28	2,05	0,74	1,20
C		2,21	1,59	0,89	0,89	1,12	14,98	0,89	0,97	2,36	1,66	1,74
D		1,97	0,97	0,50	0,27	0,89	0,74	0,43	1,36	2,90	0,81	1,82
E	1,97	1,66	3,06	1,66	0,97	3,14	0,74	0,50	0,74	1,59	1,05	1,28
F	1,43	0,74	0,97	1,28	0,97	1,36	1,51	0,97	0,97	0,58	2,36	1,82
G	1,43	1,20	0,97	0,81	0,58	2,44	1,28	0,97	0,58	2,13	1,36	0,81
H	2,83	0,43	1,43	0,35	0,81	1,90	1,28	0,89	1,59	0,74	1,82	1,51
