# Supplementary material for: Serological Insights into Infectious Agents Circulating in Lithuanian Goats
Source: Vet Sci. 2026 Jan 15;13(1):86. doi: 10.3390/vetsci13010086 (PMC12846376; doi:10.3390/vetsci13010086)
Supplement: Supplementary file 1 [file vetsci-13-00086-s001.zip › Supplementary Table S7. CAE.pdf]

CAEV results

Sample number	Farm	ELISA	OD (opt. dens.)	S/P ratio%
**1**	Farm 1	+	0,211	437,18
**2**	Farm 1	+	0,207	426,92
**3**	Farm 1	+	0,163	314,1
**4**	Farm 1	+	0,156	296,15
**5**	Farm 1	+	0,153	288,46
**6**	Farm 1	+	0,219	457,69
**7**	Farm 1	+	0,224	470,51
**8**	Farm 1	+	0,103	160,26
**9**	Farm 1	+	0,191	385,9
**10**	Farm 1	+	0,191	385,9
**11**	Farm 2	-	0,04	-1,28
**12**	Farm 2	-	0,035	-14,1
**13**	Farm 2	-	0,044	8,97
**14**	Farm 2	-	0,049	21,79
**15**	Farm 2	-	0,041	1,28
**16**	Farm 2	-	0,043	6,41
**17**	Farm 2	-	0,042	3,85
**18**	Farm 2	-	0,041	1,28
**19**	Farm 2	-	0,043	6,41
**20**	Farm 2	-	0,046	14,1
**21**	Farm 3	-	0,041	1,28
**22**	Farm3	-	0,042	3,85
**23**	Farm 3	-	0,041	1,28
**24**	Farm 3	+	0,175	344,87
**25**	Farm 3	-	0,04	-1,28
**26**	Farm 3	-	0,042	3,85
**27**	Farm 3	-	0,04	-1,28
**28**	Farm 3	+	0,2	408,97
**29**	Farm 3	-	0,041	1,28
**30**	Farm 3	+	0,21	434,62
**31**	Farm 4	-	0,042	3,85
**32**	Farm 4	-	0,041	1,28
**33**	Farm 4	-	0,038	-6,41
**34**	Farm 4	-	0,043	6,41
**35**	Farm 4	-	0,037	-8,97
**36**	Farm 4	+	0,073	83,33
**37**	Farm 4	-	0,041	1,28
**38**	Farm 4	-	0,04	-1,28
**39**	Farm 4	-	0,04	-1,28
**40**	Farm 4	-	0,042	3,85
**41**	Farm 4	+	0,082	106,41

CAEV results

**42**	Farm 5	-	0,041	1,28
**43**	Farm 5	-	0,05	24,36
**44**	Farm 5	-	0,04	-1,28
**45**	Farm 5	-	0,055	37,18
**46**	Farm 5	-	0,047	16,67
**47**	Farm 5	+	0,144	265,38
**48**	Farm 5	-	0,043	6,41
**49**	Farm 5	-	0,043	6,41
**50**	Farm 5		0,042	3,85
**51**	Farm 6	-	0,041	1,28
**52**	Farm 6	-	0,047	16,67
**53**	Farm 6	-	0,041	1,28
**54**	Farm 6	-	0,042	3,85
**55**	Farm 6	-	0,043	6,41
**56**	Farm 6	-	0,041	1,28
**57**	Farm 6	-	0,042	3,85
**58**	Farm 6	-	0,036	-11,54
**59**	Farm 6	-	0,041	1,28
**60**	Farm 6	-	0,04	-1,28
**61**	Farm 6	-	0,044	8,97
**62**	Farm 7	+	0,146	270,51
**63**	Farm 7	-	0,041	1,28
**64**	Farm 7	-	0,043	6,41
**65**	Farm 7	-	0,042	3,85
**66**	Farm 7	-	0,041	1,28
**67**	Farm 7	-	0,043	6,41
**68**	Farm 7	-	0,039	-3,85
**69**	Farm 7	+	0,216	450
**70**	Farm 7	-	0,041	1,28
**71**	Farm 7	-	0,042	3,85
**72**	Farm 8	+	3,558	228,13
**73**	Farm 8	+	3,821	245,35
**74**	Farm 8	+	OVRFLW	VALUE
**75**	Farm 8	+	OVRFLW	VALUE
**76**	Farm 8	+	OVRFLW	VALUE
**77**	Farm 8	+	OVRFLW	VALUE
**78**	Farm 8	+	1,29	79,6
**79**	Farm 8	+	1,545	96,3
**80**	Farm 8	-	0,089	0,95
**81**	Farm 8	-	0,095	1,34
**82**	Farm 8	-	0,15	4,94
**83**	Farm 8	+	OVRFLW	VALUE
**84**	Farm 9	-	0,041	1,28

CAEV results

**85**	Farm 9	+	0,145	267,95
**86**	Farm 9	+	0,145	267,95
**87**	Farm 9	+	0,197	401,28
**88**	Farm 9	+	0,07	75,64
**89**	Farm 9	-	0,041	1,28
**90**	Farm 9	-	0,041	1,28
**91**	Farm 9	+	0,214	444,87
**92**	Farm 9	+	0,194	393,59
**93**	Farm 9	+	0,145	267,95
**94**	Farm 9	-	0,087	0,82
**95**	Farm 10	-	0,043	6,41
**96**	Farm 10	-	0,042	3,85
**97**	Farm 10	-	0,04	-1,28
**98**	Farm 10	-	0,042	3,85
**99**	Farm 10	-	0,04	-1,28
**100**	Farm 10	-	0,041	1,28
**101**	Farm 10	-	0,042	3,85
**102**	Farm 10	-	0,043	6,41
**103**	Farm 10	-	0,044	8,97
**104**	Farm 10	-	0,039	-3,85
**105**	Farm 10	-	0,041	1,28
**106**	Farm 11	+	ovrflw	VALUE
**107**	Farm 11	-	0,099	1,6
**108**	Farm 11	-	0,096	1,41
**109**	Farm 11	-	0,08	0,36
**110**	Farm 11	+	3,138	200,62
**111**	Farm 11	-	0,079	0,29
**112**	Farm 11	-	0,09	1,02
**113**	Farm 11	+	1,131	69,19
**114**	Farm 11	-	0,118	2,85
**115**	Farm 11	-	0,333	16,93
**116**	Farm 12	-	0,13	3,63
**117**	Farm 12	-	0,078	0,23
**118**	Farm 12	-	0,081	0,43
**119**	Farm 12	-	0,08	0,36
**120**	Farm 12	-	0,083	0,56
**121**	Farm 12	-	0,099	1,6
**122**	Farm 12	-	0,081	0,43
**123**	Farm 12	-	0,118	2,85
**124**	Farm 12	-	0,079	0,29
**125**	Farm 12	-	0,099	1,6
**126**	Farm 12	-	0,085	0,69
**127**	Farm 12	-	0,078	0,23

CAEV results

**128**	Farm 12	-	0,085	0,69
**129**	Farm 12	-	0,083	0,56
**130**	Farm 12	-	0,211	8,94
**131**	Farm 13	-	0,107	2,13
**132**	Farm 13	-	0,075	0,03
**133**	Farm 13	-	0,087	0,82
**134**	Farm 13	-	0,083	0,56
**135**	Farm 13	-	0,079	0,29
**136**	Farm 13	-	0,107	2,13
**137**	Farm 13	-	0,081	0,43
**138**	Farm 13	-	0,097	1,47
**139**	Farm 13	-	0,085	0,69
**140**	Farm 13	-	0,087	0,82
**141**	Farm 13	-	0,117	2,78
**142**	Farm 13	-	0,087	0,82
**143**	Farm 13	-	0,165	5,93
**144**	Farm 13	-	0,125	3,31
**145**	Farm 13	-	0,092	1,15
**146**	Farm 14	-	0,117	2,78
**147**	Farm 14	-	0,079	0,29
**148**	Farm 14	-	0,081	0,43
**149**	Farm 14	-	0,309	15,36
**150**	Farm 14	-	0,121	3,05
**151**	Farm 14	-	0,081	0,43
**152**	Farm 14	+	2,511	159,56
**153**	Farm 14	-	0,097	1,47
**154**	Farm 14	-	0,079	0,29
**155**	Farm 14	-	0,119	2,91
**156**	Farm 14	-	0,383	20,2
**157**	Farm 14	-	0,106	2,06
**158**	Farm 14	-	0,078	0,23
**159**	Farm 14	-	0,1	1,67
**160**	Farm 14	-	0,128	3,5
**161**	Farm 15	-	0,178	6,78
**162**	Farm 15	-	0,085	0,69
**163**	Farm 15	-	0,131	3,7
**164**	Farm 15	-	0,138	4,16
**165**	Farm 15	-	0,083	0,56
**166**	Farm 15	-	0,081	0,43
**167**	Farm 15	-	0,081	0,43
**168**	Farm 15	-	0,108	2,19
**169**	Farm 15	-	0,107	2,13
**170**	Farm 15	-	0,165	5,93
**171**	Farm 15	-	0,143	4,49

CAEV results

**172**	Farm 15	-	0,08	0,36
**173**	Farm 16	-	0,138	6,15
**174**	Farm 16	-	0,105	3,44
**175**	Farm 16	-	0,081	1,47
**176**	Farm 16	-	0,159	7,87
**177**	Farm 16	-	0,11	3,85
**178**	Farm 16	-	0,082	1,56
**179**	Farm 16	-	0,126	5,16
**180**	Farm 16	-	0,097	2,79
**181**	Farm 16	-	0,074	0,9
**182**	Farm 16	-	0,093	2,46
**183**	Farm 16	-	0,12	4,67
**184**	Farm 16	-	0,091	2,29
**185**	Farm 16	-	0,088	2,05
**186**	Farm 16	-	0,075	0,98
**187**	Farm 16	-	0,088	2,05
**188**	Farm 16	-	0,095	2,62
**189**	Farm 17	-	0,085	1,8
**190**	Farm 17	-	0,088	2,05
**191**	Farm 17	-	0,098	2,87
**192**	Farm 17	-	0,082	1,56
**193**	Farm 17	-	0,08	1,39
**194**	Farm 17	-	0,083	1,64
**195**	Farm 17	-	0,09	2,21
**196**	Farm 17	-	0,095	2,62
**197**	Farm 17	-	0,092	2,38
**198**	Farm 17	-	0,11	3,85
**199**	Farm 17	-	0,118	4,51
**200**	Farm 17	-	0,187	10,16
**201**	Farm 17	-	0,086	1,88
**202**	Farm 17	-	0,095	2,62
**203**	Farm 17	-	0,083	1,64
**204**	Farm 18	-	0,583	42,61
**205**	Farm 18	-	0,076	1,07
**206**	Farm 18	-	0,099	2,95
**207**	Farm 18	-	0,09	2,21
**208**	Farm 18	+	1,732	136,75
**209**	Farm 18	+	OVRFLW	VALUE
**210**	Farm 18	+	OVRFLW	VALUE
**211**	Farm 18	-	0,094	2,54
**212**	Farm 18	-	0,088	2,05
**213**	Farm 18	+	3,81	307,01
**214**	Farm 18	-	0,117	4,42

CAEV results

**215**	Farm 18	-	0,104	3,36
**216**	Farm 19	-	0,102	3,2
**217**	Farm 19	-	0,226	13,36
**218**	Farm 19	-	0,123	4,92
**219**	Farm 19	-	0,088	2,05
**220**	Farm 19	-	0,133	5,74
**221**	Farm 19	-	0,091	2,29
**222**	Farm 19	-	0,389	26,71
**223**	Farm 19	-	0,068	0,41
**224**	Farm 19	-	0,082	1,56
**225**	Farm 19	-	0,089	2,13
**226**	Farm 19	-	0,105	3,44
**227**	Farm 19	-	0,075	0,98
**228**	Farm 19	-	0,096	2,7
**229**	Farm 19	-	0,102	3,2
**230**	Farm 20	-	0,093	2,46
**231**	Farm 20	-	0,147	6,88
**232**	Farm 20	-	0,142	6,47
**233**	Farm 20	-	0,085	1,8
**234**	Farm 20	-	0,079	1,31
**235**	Farm 20	+	OVRFLW	VALUE
**236**	Farm 20	-	0,12	4,67
**237**	Farm 20	+	1,786	141,17
**238**	Farm 20	+	3,92	316,02
**239**	Farm 20	+	1,719	135,68
**240**	Farm 20	+	OVRFLW	VALUE
**241**	Farm 20	+	OVRFLW	VALUE
**242**	Farm 20	-	0,077	1,15
**243**	Farm 20	-	0,087	1,97
**244**	Farm 20	+	OVRFLW	VALUE
**245**	Farm 20	+	OVRFLW	VALUE
**246**	Farm 21	-	0,144	6,64
**247**	Farm 21	-	0,102	3,2
**248**	Farm 21	-	0,09	2,21
**249**	Farm 21	-	0,09	2,21
**250**	Farm 21	-	0,097	2,79
**251**	Farm 21	-	0,076	1,07
**252**	Farm 21	-	0,094	2,54
**253**	Farm 21	-	0,086	1,88
**254**	Farm 21	-	0,126	5,16
**255**	Farm 21	-	0,099	2,95
**256**	Farm 21	-	0,081	1,47
**257**	Farm 21	-	0,097	2,79
**258**	Farm 21	-	0,085	1,8

CAEV results

**259**	Farm 21	-	0,082	1,56
**260**	Farm 21	-	0,103	3,28
**261**	Farm 21	-	0,113	4,1
**262**	Farm 3	-	0,106	2,74
**263**	Farm 3	-	0,104	2,6
**264**	Farm 3	-	0,077	0,75
**265**	Farm 3	+	3,035	203,22
**266**	Farm 3	-	0,08	0,96
**267**	Farm 3	-	0,123	3,9
**268**	Farm 3	-	0,091	1,71
**269**	Farm 3	+	3,686	247,78
**270**	Farm 3	-	0,084	1,23
**271**	Farm 3	-	0,34	18,75
**272**	Farm 3	+	OVRFLW	VALUE
**273**	Farm 3	-	0,082	1,1
**274**	Farm 3	-	0,327	17,86
**275**	Farm 3	+	1,733	114,1
**276**	Farm 3	-	0,077	0,75
**277**	Farm 3	+	2,332	155,1
**278**	Farm 3	-	0,111	3,08
**279**	Farm 3	+	3,446	231,35
**280**	Farm 22	-	0,083	1,16
**281**	Farm 22	-	0,133	4,59
**282**	Farm 22	-	0,078	0,82
**283**	Farm 22	-	0,179	7,73
**284**	Farm 22	-	0,087	1,44
**285**	Farm 22	+	1,28	83,09
**286**	Farm 22	-	0,096	2,05
**287**	Farm 22	-	0,093	1,85
**288**	Farm 22	-	0,096	2,05
**289**	Farm 22	-	0,072	0,41
**290**	Farm 22	-	0,085	1,3
**291**	Farm 22	-	0,139	5
**292**	Farm 23	-	0,095	1,98
**293**	Farm 23	-	0,088	1,51
**294**	Farm 23	-	0,081	1,03
**295**	Farm 23	-	0,101	2,4
**296**	Farm 23	-	0,092	1,78
**297**	Farm 23	-	0,11	3,01
**298**	Farm 23	-	0,085	1,3
**299**	Farm 23	-	0,077	0,75
**300**	Farm 23	+	OVRFLW	VALUE
**301**	Farm 23	-	0,165	6,78
**302**	Farm 23	-	0,171	7,19

CAEV results

**303**	Farm 23	-	0,147	5,54
**304**	Farm 23	-	0,083	1,16
**305**	Farm 23	-	0,105	2,67
**306**	Farm 23	-	0,075	0,62
**307**	Farm 24	-	0,187	8,28
**308**	Farm 24	-	0,098	2,19
**309**	Farm 24	-	0,177	7,6
**310**	Farm 24	-	0,166	6,84
**311**	Farm 24	-	0,094	1,92
**312**	Farm 24	+	OVRFLW	VALUE
**313**	Farm 24	+	OVRFLW	VALUE
**314**	Farm 24	-	0,706	43,81
**315**	Farm 24	-	0,085	1,3
**316**	Farm 24	-/+	0,896	56,81
**317**	Farm 24	+	OVRFLW	VALUE
**318**	Farm 24	-	0,134	4,65
**319**	Farm 24	-	0,089	1,57
**320**	Farm 24	+	3,764	253,11
**321**	Farm 24	+	OVRFLW	VALUE
**322**	Farm 24	-	0,213	10,06
**323**	Farm 24	-	0,12	3,7
**324**	Farm 24	-	0,082	1,1
**325**	Farm 24	-	0,235	11,57
**326**	Farm 25	-	0,076	0,89
**327**	Farm 25	-	0,125	5,06
**328**	Farm 25	+	2,121	174,64
**329**	Farm 25	-	0,144	6,67
**330**	Farm 25	-	0,102	3,1
**331**	Farm 25	-	0,482	35,39
**332**	Farm 25	-	0,085	1,66
**333**	Farm 25	+	2,751	228,16
**334**	Farm 25	-	0,068	0,21
**335**	Farm 25	-	0,07	0,38
**336**	Farm 25	-	0,073	0,64
**337**	Farm 26	-	0,078	1,06
**338**	Farm 26	-	0,093	2,34
**339**	Farm 26	-	0,093	2,34
**340**	Farm 26	-	0,083	1,49
**341**	Farm 27	-	0,078	1,06
**342**	Farm 27	-	0,262	16,69
**343**	Farm 27	-	0,094	2,42
**344**	Farm 27	-	0,079	1,15
**345**	Farm 27	-	0,113	4,04

CAEV results

**346**	Farm 27	-	0,109	3,7
**347**	Farm 27	+	3,304	275,15
**348**	Farm 27	-	0,074	0,72
**349**	Farm 27	-	0,11	3,78
**350**	Farm 27	-	0,182	9,9
**351**	Farm 27	+	1,338	108,11
**352**	Farm 28	-	0,16	8,03
**353**	Farm 28	-	0,099	2,85
**354**	Farm 28	-	0,084	1,57
**355**	Farm 28	-	0,105	3,36
**356**	Farm 29	-	0,083	1,49
**357**	Farm 29	-	0,072	0,55
**358**	Farm 29	-	0,077	0,98
**359**	Farm 29	-	0,093	2,34
**360**	Farm 29	-	0,149	7,09
**361**	Farm 29	+	3,225	268,44
**362**	Farm 29	+	2,973	247,03
**363**	Farm 29	+	OVRFLW	VALUE
**364**	Farm 29	-	0,074	0,72
**365**	Farm 30	-	0,185	10,15
**366**	Farm 30	-	0,086	1,74
**367**	Farm 30	-	0,104	3,27
**368**	Farm 30	-	0,086	1,74
**369**	Farm 28	-	0,186	10,24
**370**	Farm 27	-	0,081	1,32
**371**	Farm 18	-	0,073	1,51
**372**	Farm 18	+	2,761	389,11
**373**	Farm 18	-	0,099	5,26
**374**	Farm 18	-	0,087	3,53
**375**	Farm 3	+	3,537	501,01
**376**	Farm 3	+	2,44	342,83
**377**	Farm 3	-	0,076	1,95
**378**	Farm 3	-	0,081	2,67
**379**	Farm 14	+	2,901	409,3
**380**	Farm 7	-	0,082	2,81
